# Tangled contacts: mitochondrial interactions with cellular organelles in acute pancreatitis

**DOI:** 10.3389/fgstr.2026.1802268

**Published:** 2026-04-10

**Authors:** Serge Chooklin, Serhii Chuklin

**Affiliations:** Surgical Center, Saint Paraskeva Medical Center, Lviv, Ukraine

**Keywords:** acute pancreatitis, inter-organellar interactions, mitochondria, mitochondria-associated membranes, pancreatic acinar cells

## Abstract

**Objective:**

Mitochondria in pancreatic acinar cells function as central hubs integrating calcium signaling, ATP production, redox balance, autophagy, secretion, and cell-death regulation through dynamic interactions with other organelles.

**Aim:**

To summarize current evidence on mitochondria–organelle interactions in pancreatic acinar cells and their relevance to acute pancreatitis.

**Methods:**

We performed a narrative review of experimental and translational studies addressing mitochondrial interactions with the endoplasmic reticulum, lysosomes, autophagosomes, peroxisomes, the cytoskeleton, plasma membrane, nucleus, lipid droplets, and secretory granules in pancreatic acinar cells and experimental acute pancreatitis.

**Results:**

Mitochondria–endoplasmic reticulum contacts emerged as major determinants of pathological Ca^2+^ transfer, mitochondrial depolarization, and ATP depletion. Impaired crosstalk with lysosomes and autophagosomes disrupted mitophagy and favored the persistence of dysfunctional mitochondria, defective vacuolar processing, and inflammatory amplification. Altered functional coupling with peroxisomes and lipid droplets intensified oxidative stress, fatty-acid disequilibrium, and lipotoxic injury, particularly in metabolically unfavorable settings. Disturbed interactions with the cytoskeleton and plasma membrane impaired mitochondrial positioning, local Ca^2+^ buffering, and the spatial organization of stimulus–secretion coupling. Mitochondria-to-nucleus signaling promoted stress-responsive and proinflammatory transcriptional programs, while mitochondrial failure in the apical secretory region indirectly facilitated defective exocytosis and premature zymogen activation. Collectively, these alterations shifted acinar cells from adaptive stress responses toward necrosis, local pancreatic damage, systemic inflammation, and organ failure.

**Conclusions:**

Mitochondria-associated inter-organellar networks are integral to acinar-cell homeostasis and critically influence the initiation and progression of acute pancreatitis. Their selective stabilization may represent a mechanistically grounded therapeutic direction.

## 1 Introduction

Acute pancreatitis (AP) remains a major challenge in contemporary gastroenterology and surgery because of its highly variable clinical course, ranging from mild and self-limiting inflammation to severe forms associated with multiple organ failure and high mortality. Epidemiological data indicate that the incidence of AP continues to rise worldwide, emphasizing its substantial clinical and socioeconomic burden (1). Despite progress in diagnostic methods and intensive care strategies, the outcomes of severe AP remain unsatisfactory, underscoring the need for a deeper understanding of the mechanisms responsible for pancreatic injury and the development of systemic complications (2).

The pathogenesis of AP is multifactorial and involves a complex interplay of premature intracellular enzyme activation, calcium overload, oxidative stress, inflammatory signaling, immune dysregulation, and mitochondrial damage in pancreatic acinar cells (PACs) (3, 4). Although mitochondria have traditionally been viewed primarily as the main source of cellular ATP, they are now recognized as multifunctional organelles that regulate calcium signaling, redox homeostasis, stress adaptation, cell death pathways, and intracellular homeostasis. In highly specialized secretory cells such as PACs, these roles are particularly important because cellular viability and secretory activity depend on tight coordination between energy production and intracellular signaling processes (4, 5). Mitochondrial activity in such cells is inseparable from its interactions with other intracellular compartments, including the endoplasmic reticulum (ER), lysosomes, the cytoskeleton, the plasma membrane, the nucleus, lipid droplets, peroxisomes, and secretory granules (6). The structural and functional integrity of these interactions is crucial for maintaining normal acinar-cell function, whereas their disruption may substantially contribute to pancreatic injury during AP (7).

Recent advances in cell biology increasingly support the concept that mitochondria should not be regarded as isolated bioenergetic organelles, but rather as dynamic signaling platforms embedded within an integrated intracellular network. In this context, particular attention has been drawn to the idea of a “mitochondrial hub,” according to which mitochondria coordinate cellular stress responses through highly organized contact sites with other organelles (8). In PACs, this perspective appears especially relevant because these cells combine intense secretory activity with marked vulnerability to disturbances in calcium and redox balance. Under physiological conditions, coordinated mitochondria–ER interactions ensure localized Ca2+ transfer, activation of tricarboxylic acid cycle enzymes, maintenance of ATP synthesis, and prevention of excessive cytosolic calcium accumulation, thereby supporting controlled exocytosis and cellular homeostasis (9).

During AP, these finely balanced inter-organellar relationships become disrupted. Alterations in mitochondria-associated ER membranes, excessive production of reactive oxygen species, impaired autophagic flux, disturbed calcium transport, mitochondrial fragmentation, and activation of inflammatory pathways collectively drive progressive organellar dysfunction (10–12). These changes may result in bioenergetic failure, defective organelle quality control, activation of apoptotic, necroptotic, and inflammatory cascades, and release of damage-associated molecular patterns that intensify sterile inflammation and contribute to both local and systemic injury (13, 14). In particular, defective coordination between mitochondria, lysosomes, and autophagosomes compromises mitophagy, leading to the accumulation of damaged mitochondria and further amplification of oxidative and inflammatory stress. Disruption of mitochondrial interactions with the cytoskeleton may also impair intracellular trafficking and the spatial distribution of energy in areas with high metabolic demand.

Within this framework, further consideration of AP through the lens of inter-organellar communication may offer an important conceptual advance. Such an approach creates an opportunity to move beyond the description of isolated subcellular abnormalities and instead examine how disturbances at specific organelle contact sites evolve into integrated cellular failure. Particular interest may lie in clarifying the sequence by which early defects at ER–mitochondria interfaces are followed by redox amplification, lysosomal dysfunction, altered lipid and peroxisomal buffering, inflammatory escalation, and progressive loss of secretory polarity. Viewing these processes as components of a coordinated intracellular network may improve understanding of how local organelle-level damage is translated into whole-cell dysfunction and, ultimately, systemic disease manifestations. It may also provide a more informative basis for identifying stage-specific biomarkers and for prioritizing therapeutic targets according to the temporal dynamics of AP rather than according to isolated downstream events alone.

Accordingly, the present review examines current evidence on mitochondria–organelle interactions in pancreatic acinar cells and considers how an integrated intracellular network perspective may deepen understanding of AP pathogenesis and support future progress in biomarker research and therapeutic strategy development.

## 2 Methods

### 2.1 Study design and scope

This article is a narrative review of contemporary scientific literature devoted to the inter-organellar interactions of mitochondria in PACs and their role in the pathogenesis of AP. The focus was on mechanistic and experimental evidence describing mitochondrial–ER contacts, mitochondria–lysosome crosstalk, interactions with the cytoskeleton, PM, nucleus, LDs, peroxisomes, and secretory granules, as well as integrative concepts such as the “mitochondrial hub” in PACs.

### 2.2 Literature search strategy and depth of search

**Figure 1:**
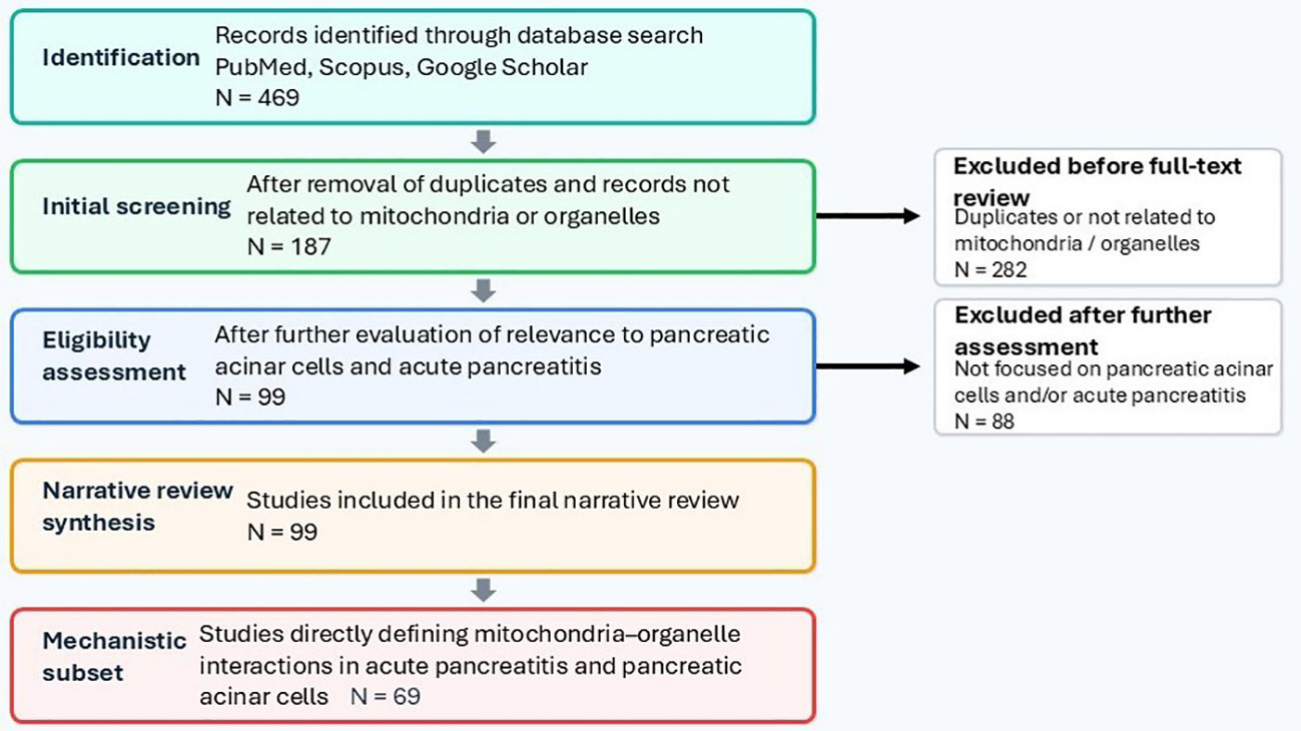
Literature search and study selection flow.

A structured literature search was performed using the international electronic databases PubMed, Scopus, and Google Scholar (**Figure 1**). The search covered all records available in these databases from their inception to December 2025, with particular emphasis on publications from 2010 to 2025, when most experimental work on mitochondrial integration in AP has been published. The following combinations of keywords and Medical Subject Headings (MeSH) terms were used: “pancreatic acinar cells,” “acute pancreatitis,” “mitochondria,” “organelle interactions,” “mitochondria–ER contact,” “mitochondria-associated membranes,” “mitophagy,” “autophagy,” “oxidative stress,” “redox signaling,” “lipid droplets,” “cytoskeleton,” “lysosomes,” and “peroxisomes.” The reference lists of key articles were additionally screened to identify further relevant primary studies (“snowball” search).

### 2.3 Inclusion and exclusion criteria

The following inclusion criteria were applied:

1. Type of study: original experimental, translational, or clinical research articles (*in vitro*, ex vivo, or *in vivo* models) providing data on mitochondrial function and/or inter-organellar interactions in the pancreas.
2. Biological focus: studies investigating PACs, whole pancreatic tissue, or experimental models of AP in which mitochondrial dysfunction, inter-organellar contact sites, mitophagy/autophagy, redox signaling, or calcium homeostasis were directly assessed.
3. Outcome domain: articles reporting structural, functional, biochemical, or molecular data relevant to mitochondrial–ER, mitochondrial–lysosome, mitochondrial–cytoskeleton, mitochondrial–PM, mitochondrial–nucleus, mitochondrial–lipid droplet, or mitochondrial–peroxisome interactions as well as their impact on AP initiation, progression, and complications.

The exclusion criteria were:

1. Studies not involving the pancreas or PACs (for example, studies limited to other organs or cancer cell lines without direct relevance to AP pathogenesis).
2. Conference abstracts, theses, case reports, editorials, and commentaries without sufficient methodological detail or primary data.
3. Papers dealing exclusively with clinical epidemiology or treatment of AP without mechanistic information on mitochondria or organelle crosstalk.

High-quality narrative and systematic reviews on mitochondrial biology, redox signaling, or AP were not excluded *a priori*; they were used to contextualize and interpret primary experimental findings but were not treated as primary evidence when synthesizing mechanistic conclusions.

### 2.4 Study selection and data handling

The identified records were screened by title and abstract to assess their relevance to mitochondrial dysfunction and inter-organellar integration in AP. Potentially eligible articles were evaluated in the full text. For each included study, information was qualitatively extracted from the experimental model (*in vitro* vs. *in vivo*, method of AP induction), organellar structures investigated, key molecular pathways (e.g., Ca^2+^ handling, mitophagy, inflammasome activation, redox signaling), and main findings related to mitochondrial–organelle crosstalk. The extracted data were also organized into a structured summary table including model type, species, organelle interaction studied, and the main mitochondrial phenotype, to facilitate comparison across the heterogeneous body of experimental evidence. Because of the substantial heterogeneity of the experimental designs, outcome measures, and mechanistic endpoints, no formal quantitative synthesis (meta-analysis) or structured risk-of-bias scoring was performed. Instead, the findings were qualitatively integrated and complemented by tabulated study-level characteristics in relation to the overarching concept of the mitochondrial hub in PACs.

### 2.5 Rationale for a narrative review

A narrative review design was selected because the goal of this article is conceptual integration rather than formal effect-size synthesis. The literature on mitochondria–organelle crosstalk in AP is highly heterogeneous with respect to model systems, injury triggers, imaging methods, molecular endpoints, and temporal windows, which limits direct comparability and makes quantitative pooling inappropriate. A narrative structure therefore allowed us to organize the field around a mechanistic question - how mitochondria-centered contact failure reshapes acinar-cell injury - and to distinguish between well-supported findings, extrapolated mechanisms, unresolved controversies, and translational gaps. This format also made it possible to compare early initiating events with later amplifying processes and to translate that hierarchy into a clinically oriented framework for biomarker discovery and therapeutic timing.

## 3 Interaction of mitochondria with other cellular structures in acute pancreatitis

**Figure 2:**
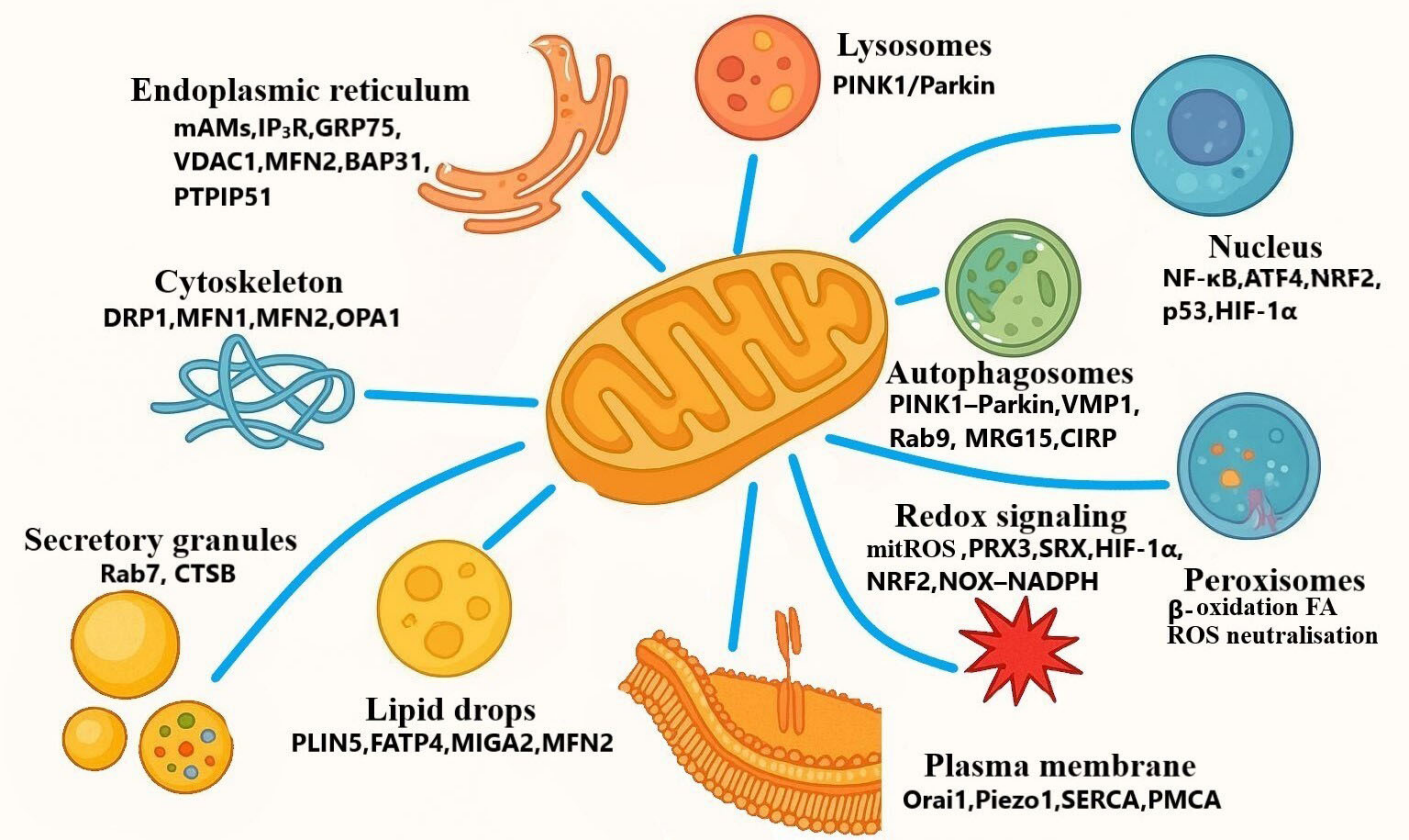
Mitochondria-centered inter-organellar interaction map in pancreatic acinar cells and acute pancreatitis.

Mitochondria are not isolated organelles; in pancreatic acinar cells (PACs), they function within an integrated intracellular network that connects early calcium dysregulation with downstream redox disturbances, lysosomal-autophagic impairment, cytoskeletal disorganization, secretory dysfunction, nuclear responses, lipid imbalance, and inflammatory activation. In view of this continuity, the following discussion traces these events as a mechanistic cascade, starting with defective ER–mitochondria calcium coupling, continuing through redox injury and impaired mitochondria–lysosome quality control, and then considering interfaces that govern polarity, metabolism, transcriptional adaptation, and inflammatory amplification. Under physiological conditions, these interactions sustain calcium homeostasis, redox balance, proper protein folding, and cellular stress adaptation. In AP, however, disruption of inter-organellar communication converts this coordinated network into a feed-forward system that intensifies mitochondrial dysfunction and acinar-cell injury (**Figure 2**). The representative studies included in this review are summarized in **Table 1**, whereas the principal mechanistic features of mitochondria–organelle interactions are integrated in **Table 2**.

**Table 1:** Structured summary of representative studies on mitochondria–organelle interactions in pancreatic acinar cells and experimental acute pancreatitis.

Model type	Species/material	Organelle interaction studied	Main mitochondrial phenotype	References
*In vitro*/mechanistic experimental studies	Pancreatic acinar cells	Endoplasmic reticulum–mitochondria Ca^2+^ coupling (MAM-related signaling)	Stimulus-coupled mitochondrial Ca^2+^ uptake, activation of oxidative metabolism, increased NADH production; under pathological conditions, Ca^2+^ overload and early bioenergetic stress	(9, 15, 17)
*In vitro*/*in vivo* experimental AP models	Predominantly rodent pancreatic tissue/acinar cells	ER–mitochondria structural and functional integration	Mitochondrial Ca^2+^ overload, dissipation of ΔΨm, mPTP opening, ATP depletion, impaired oxidative phosphorylation	(18–23)
Experimental mechanistic studies and AP models	Acinar-cell and rodent systems	Mitochondrial redox signaling with ER, peroxisomes, plasma membrane channels, and nucleus	Excess mtROS generation, redox imbalance, mitochondrial depolarization, promotion of inflammatory signaling and regulated cell death	(24–34)
*In vivo*/ex vivo AP models	Mainly rodent pancreas and pancreatic acinar cells	Mitochondria–lysosome/autophagy–mitophagy axis	Accumulation of damaged and depolarized mitochondria, defective clearance of mitochondrial remnants, persistent ROS production, energetic failure	(10, 11, 35–37)
Experimental cellular and animal studies	Pancreatic acinar cells/rodent AP models	Mitochondria–cytoskeleton crosstalk	Mitochondrial fragmentation, loss of ΔΨm, impaired mitochondrial positioning and trafficking, reduced ATP production	(35, 37–39)
*In vitro*/*in vivo* experimental studies	Pancreatic acinar cells and rodent AP models	Mitochondria–plasma membrane Ca^2+^ microdomains/SOCE-related coupling	Mitochondrial Ca^2+^ overload, mPTP opening, ΔΨm loss, suppression of ATP synthesis, failure of Ca^2+^ buffering	(16, 17, 40–43)
Mechanistic experimental studies	Pancreatic and extra-pancreatic experimental systems relevant to AP	Mitochondria–nucleus retrograde signaling	ATP depletion, mtROS overproduction, release of mitochondrial DAMPs, altered mitochondrial quality control and stress susceptibility	(8, 26, 31, 44–55)
Experimental metabolic and AP models	Mainly rodent pancreatic tissue/acinar cells	Mitochondria–lipid droplet interactions	Mitochondrial swelling and fragmentation, lipotoxic mitochondrial injury, oxidative stress, impaired mitochondrial lipid handling	(56–64)
Experimental acinar-cell physiology and AP models	Pancreatic acinar cells/rodent pancreas	Mitochondria–secretory granule coupling	Local energetic failure in the apical region, mitochondrial Ca^2+^ overload, collapse of perigranular buffering, facilitation of premature zymogen activation and necrosis	(17, 65–68)
*In vivo*/*in vitro* AP models	Predominantly mouse/rat pancreas and acinar cells	Mitochondria–autophagosome interaction during mitophagy	Removal of damaged mitochondria when effective; if impaired, persistence of dysfunctional mitochondria, ROS accumulation, bioenergetic deterioration	(35, 69–72)
Rodent experimental AP models	Mitochondria–peroxisome crosstalk	Pancreatic tissue	Decreased ΔΨm, increased ROS, impaired redox homeostasis, mitochondrial dysfunction associated with disturbed fatty-acid and peroxide metabolism	(60, 61, 73–79)

**Table 2:** Key mitochondrial–organelle interactions in pancreatic acinar cells.

Organelle partner	Structural platform/contact site	Main molecular players (examples)	Physiological functions (in acinar cells)	Pathological alterations in acute pancreatitis	Consequences for acinar cell and AP phenotype
Endoplasmic reticulum (ER)	Mitochondria-associated membranes (MAMs); close ER–mitochondria apposition	IP_3_ receptors (IP_3_R), ryanodine receptors (RyR), VDAC1, MCU complex, GRP75, MFN2, SERCA	Local Ca^2+^ transfer from ER to mitochondria; tight coupling of stimulus–secretion; regulation of ATP supply; coordination of protein synthesis and folding with energy status; integration of ER stress and mitochondrial responses	Sustained pathological Ca^2+^ release from ER; Ca^2+^ overload of mitochondria; dissipation of ΔΨm and opening of mPTP; uncoupling of oxidative phosphorylation; amplification of ER stress and unfolded protein response (UPR)	Loss of ATP production; switch from controlled secretion to premature zymogen activation; necrotic cell death; release of DAMPs; amplification of intra-pancreatic inflammation and transition to severe AP
Lysosomes/autophagosomes	Autophagolysosomes; mitochondria–lysosome contact sites	LC3, p62/SQSTM1, LAMP1/2, cathepsins, TFEB, PINK1, Parkin, DRP1	Basal turnover of damaged mitochondria (mitophagy); degradation of secretory granules and misfolded proteins; maintenance of organelle quality control and cellular homeostasis	Impaired autophagic flux; accumulation of autophagic vacuoles; defective mitophagy; lysosomal membrane permeabilization; cathepsin leakage; failure to remove dysfunctional mitochondria	Persistence of ROS-producing, depolarized mitochondria; enhanced necrosis and trypsinogen activation; worsening of local pancreatic injury; promotion of systemic inflammatory response
Zymogen granules/secretory pathway	Golgi–granule–plasma membrane secretory axis; proximity of granules to mitochondria and ER	SNARE proteins, Rab GTPases, syntaxins, synaptotagmins, Ca^2+^ channels, cytoskeletal motors	Coupling of mitochondrial ATP production and local Ca^2+^ signals to regulated exocytosis of digestive enzymes; maintenance of polarized secretion into the ductal lumen	Mislocalization of granules; disruption of apical–basal polarity; Ca^2+^-dependent premature activation of zymogens within the cell; impaired exocytosis; aberrant fusion with lysosomes	Intracellular activation of proteases; autodigestion of acinar cells; extensive necrosis; propagation of inflammatory cascade and pancreatic necrosis
Plasma membrane	Microdomains enriched in Ca^2+^ entry channels; regions close to subplasmalemmal mitochondria	ORAI1, STIM1, TRP channels, NCX, Na^+^/K^+^-ATPase, Na^+^ channels; cortical actin; subplasmalemmal mitochondria	Store-operated Ca^2+^ entry (SOCE); shaping of Ca^2+^ signals; rapid buffering of incoming Ca^2+^ by nearby mitochondria; coupling of extracellular stimuli to secretion	Hyperactivation of SOCE; impaired mitochondrial buffering due to ΔΨm loss; uncontrolled cytosolic Ca^2+^ plateaus; cell swelling and membrane rupture	Loss of membrane integrity; uncontrolled enzyme release; necrotic cell death; liberation of pro-inflammatory mediators and worsening of local and systemic inflammation
Cytoskeleton (microtubules, actin, intermediate filaments)	Mitochondria attached to microtubules and actin filaments; positioning relative to ER, granules, and plasma membrane	Tubulin, actin, kinesins, dyneins, myosins, vimentin; mitochondrial adaptor proteins (Miro, TRAK)	Spatial organization and transport of mitochondria; maintenance of acinar polarity; directed trafficking of zymogen granules; regulation of mitochondrial fission–fusion distribution	Cytoskeletal disassembly and fragmentation; impaired mitochondrial motility; loss of polarized distribution of organelles; collapse of apical–basal polarity	Mismatch between energy supply and local demand; disturbed secretory pathway; increased susceptibility to Ca^2+^ overload and necrosis; facilitation of cell detachment and tissue disorganization
Nucleus	Mitochondria positioned near nuclear envelope; retrograde signaling pathways	ROS-sensitive transcription factors (NF-κB, AP-1), NRF2, PGC-1α, HIF-1α; mtDAMP-induced pathways	Integration of mitochondrial metabolic status with gene expression; induction of antioxidant defenses; regulation of inflammatory and stress-response genes; mitochondrial biogenesis	Excess ROS and mtDAMPs drive maladaptive activation of inflammatory transcription factors; impaired induction of protective antioxidant and mitophagy programs; possible DNA damage	Up-regulation of pro-inflammatory cytokines and chemokines; insufficient cytoprotective responses; progression from local pancreatic injury to systemic inflammation and multiple organ dysfunction
Lipid droplets	Physical apposition of mitochondria and lipid droplets; shared enzymes of lipid metabolism	ATGL, HSL, PLIN proteins, CPT1, acyl-CoA synthetases; enzymes of β-oxidation	Storage and mobilization of fatty acids; provision of substrates for mitochondrial β-oxidation and ATP production; buffering of excess lipids	Lipotoxicity due to excessive liberation of long-chain fatty acids; overload of mitochondrial β-oxidation; ROS generation; formation of fatty acid ethyl esters (FAEEs) in alcohol-related AP	Mitochondrial depolarization and Ca^2+^ deregulation; exacerbation of necrosis and local inflammation; contribution to clinically more severe forms of AP, especially in hypertriglyceridemia and alcohol-induced disease
Peroxisomes	Peroxisome–mitochondria contacts in regions of shared lipid and ROS metabolism	PEX proteins, catalase, oxidases, enzymes of very-long-chain fatty acid oxidation	Detoxification of hydrogen peroxide; β-oxidation of very-long-chain fatty acids; cooperative control of cellular redox state with mitochondria	Overload of ROS-generating pathways; insufficient peroxisomal detoxification; spillover of H_2_O_2_ and other ROS; disturbed lipid metabolism	Enhanced oxidative damage to mitochondrial membranes and mtDNA; promotion of mitochondrial dysfunction and cell death; amplification of inflammatory signaling in AP

### 3.1 Mitochondria–ER contacts

The functional interaction between the ER and mitochondria in PACs is mediated by specialized zones of inter-organellar contact (MAMs), which are crucial for inter-organellar Ca^2+^ transfer, the regulation of energy metabolism, and the maintenance of cellular homeostasis. Experimental models indicate that these contacts facilitate the initiation of Ca^2+^-dependent exocytosis, support efficient ATP production, and limit the excessive accumulation of Ca^2+^ in the cytosol, thereby preserving cell viability (15).

Under physiological conditions, calcium signals generated by the ER in response to stimulation of acetylcholine (ACh) or CCK receptors are transmitted to mitochondria via a sequence of protein structures: IP_3_R (inositol trisphosphate receptors) channels on the ER, the chaperone GRP75 (75-kDa glucose-regulated protein), the channel VDAC1 (voltage-dependent anion channel 1) on the outer mitochondrial membrane (OMM), and the mitochondrial calcium uniporter (MCU) on the inner mitochondrial membrane (IMM). Such coordinated signaling ensures rapid Ca^2+^ uptake into the mitochondrial matrix, activation of TCA cycle enzymes, and stimulation of ATP synthesis (9). Perigranularly localized mitochondria form a buffering zone that limits local Ca^2+^ fluctuations, promotes controlled secretion, and protects the cell from the spread of pathological calcium signals (16). Calcium accumulation in the mitochondria is accompanied by an increase in the level of NADH, which is required for the function of the electron transport chain (ETC) and maintenance of intracellular energy production (17). These data strongly support the concept of MAMs as permissive platforms for efficient stimulus–metabolism coupling, but they are largely based on indirect readouts (Ca^2+^ imaging, NADH autofluorescence, ATP sensors) rather than direct dynamic visualization of individual MAM microdomains, and thus cannot fully resolve the causality between structural contacts and functional responses. Under pathological conditions, however, sustained Ca^2+^ transfer from the ER to mitochondria is consistently associated with mitochondrial injury and is widely considered a candidate early pathogenic event, although the temporal sequence remains incompletely resolved.

The physical and functional intertwining of the ER and mitochondria determines their roles as integrators of cellular signaling, including inflammation, autophagy, and metabolic control (18). Under conditions of ER stress, altered ER–mitochondria coupling has been associated with uncontrolled calcium influx into mitochondria, opening of the mitochondrial permeability transition pore (mPTP), loss of mitochondrial membrane potential (ΔΨm), reduced ATP production, activation of apoptosis, and enhancement of the inflammatory response (19). Mitochondrial Ca^2+^ overload is closely associated with mPTP opening, dissipation of ΔΨm, and impairment of oxidative phosphorylation, thereby placing bioenergetic failure among the earliest direct consequences of abnormal ER–mitochondria Ca^2+^ signaling. By contrast, inflammatory activation and progressive oxidative injury appear to function predominantly as downstream processes that exacerbate mitochondrial dysfunction and cell damage after the initial loss of calcium homeostasis has occurred. However, it remains unclear whether MAM disruption is an early initiating event or a secondary consequence of mitochondrial depolarization and bioenergetic failure. Many studies have relied on static ultrastructural analyses or proximity-based assays performed at single time points, which provide only snapshots of a highly dynamic interface. Longitudinal *in vivo* imaging of MAM dynamics in AP is lacking, making temporal relationships between ER stress, MAM remodeling, and cell death largely inferential.

Protein complexes that provide structural and functional integration of these organelles include IP3R, GRP75, VDAC1, mitofusin-2 (MFN2), BAP31 (B-cell receptor–associated protein 31), and PTPIP51 (protein tyrosine phosphatase–interacting protein 51). They create a platform for efficient calcium transfer and participate in signaling pathways that regulate cell viability (19–21). However, much of the mechanistic work dissecting individual tethers (e.g., MFN2, VAPB–PTPIP51, BAP31–FIS1) has been performed in neurons, cancer cell lines, and non-pancreatic tissues (19–21). Extrapolation of these findings to PACs is reasonable, but must be made cautiously, given tissue-specific differences in organelle architecture, secretory load, and calcium signaling patterns. Moreover, genetic models often involve global knockdown or overexpression, so systemic effects and compensatory adaptations in other organs may confound pancreas-specific conclusions. These limitations make it difficult to define the exact temporal contribution of individual tethering proteins to the early and late phases of acinar cell injury.

In AP, activation of the IP_3_R–STIM1–Orai1 pathway promotes prolonged elevation of cytosolic Ca^2+^, leading to mitochondrial calcium overload and injury (22). Energy deficiency prevents the proper function of sarcoplasmic/ER Ca^2+^-ATPase (SERCA) and PM Ca^2+^-ATPase (PMCA) calcium pumps, thus establishing a vicious cycle of ionic imbalance (4). Simultaneously, mitochondria, in cooperation with the ER and extracellular matrix, act as calcium buffers, maintaining intracellular Ca^2+^ homeostasis (23). In this setting, Ca^2+^ overload appears to initiate mitochondrial damage, whereas ATP depletion further amplifies calcium dysregulation by compromising ATP-dependent Ca^2+^ extrusion and reuptake mechanisms. Importantly, the absence of substantial protection in experimental AP after genetic MCU ablation does not negate the pathogenic role of mitochondrial calcium overload, but rather indicates that MCU-dependent matrix Ca^2+^ uptake is not the sole determinant of disease severity. ER Ca^2+^ release, sustained store-operated Ca^2+^ entry, and persistent cytosolic Ca^2+^ elevation can continue to damage PACs even when mitochondrial Ca^2+^ entry through MCU is reduced, because calcium-dependent digestive enzyme activation, PM injury, and failure of ATP-dependent ion pumps remain operative. In addition, acinar cells retain a partial capacity to support ATP homeostasis through glycolytic pathways, which may transiently compensate for impaired mitochondrial stimulus–metabolism coupling and thereby attenuate the overall phenotypic effect of MCU loss. A notable paradox is that genetic ablation of the MCU, which markedly reduces mitochondrial Ca^2+^ uptake and stimulus–metabolism coupling in PACs, does not significantly decrease the severity of experimental AP (17). This apparent discrepancy therefore supports an integrative model in which mitochondrial Ca^2+^ overload remains mechanistically important, but acts within a broader and partially redundant injury network that also includes extramitochondrial Ca^2+^ sources, residual bioenergetic support from glycolysis, ROS generation, and inflammatory amplification. It also suggests that once mitochondrial injury has been initiated, its further progression may become increasingly independent of MCU itself owing to self-sustaining feed-forward mechanisms such as mPTP opening, oxidative stress, defective Ca^2+^ extrusion, and inflammatory signaling.

Computational models of complex Ca^2+^ dynamics incorporating store-operated Ca^2+^ entry and MAM geometry further underscore the nonlinear behavior of this system and its sensitivity to small changes in channel density, ER store content, or mitochondrial buffering capacity (15). While such models provide valuable mechanistic insights, they typically rely on parameter sets calibrated to specific experimental conditions and do not yet account for inter-animal variability, age- or sex-related differences, or the heterogeneous microenvironment of the inflamed pancreas. Taken together, available data indicate that abnormal ER-to-mitochondria Ca^2+^ transfer and mitochondrial Ca^2+^ overload are early pathogenic events, whereas oxidative stress, inflammatory activation, and impaired clearance of damaged mitochondria contribute mainly to propagation and amplification of acinar cell injury. Accordingly, mitochondrial calcium overload is best interpreted as a central early driver of injury, but not as an isolated or universally rate-limiting event, because compensatory bioenergetic pathways and parallel Ca^2+^-dependent injury mechanisms can preserve disease progression despite attenuation of MCU-mediated mitochondrial Ca^2+^ uptake.

### 3.2 Mitochondrial redox signaling as a means of inter-organellar communication

If ER–mitochondria calcium coupling represents an early trigger of injury, redox signaling is one of the main mechanisms by which that injury is propagated to the rest of the organelle network. Redox signaling is an important mechanism of cellular adaptation to stress, tightly coupled to mitochondrial activity and integrated into inter-organellar communication networks. Under physiological conditions, mitochondrial ROS (mtROS) act as short-lived secondary messengers that modulate protein function at the level of the ER, peroxisomes, plasma-membrane channels, and nuclear transcription factors, thereby coordinating proliferation, autophagy, and apoptosis. In AP, however, this finely tuned system shifts toward pathological oxidative stress, with disturbed redox homeostasis promoting excessive ROS production and propagation of organellar injury in PACs (24).

Mitochondria are a major intracellular ROS source in AP, particularly at complexes I and III of the ETC. Excessive mtROS generation accompanies mitochondrial dysfunction and promotes the transition from adaptive stress signaling to irreversible cell injury and death (12, 25). Within the framework of organelle crosstalk, mtROS are especially relevant because they extend mitochondrial stress to other compartments, including the ER and nucleus, and contribute to the loss of coordinated organellar homeostasis. In addition, redox regulation involving the mitochondrial peroxiredoxin-3 (PRX3)–sulfiredoxin (SRX) system appears to participate in controlling oxidative stress-induced necroptotic responses in AP (26). Thus, mtROS should be viewed not only as mediators of mitochondrial damage but also as signals that disseminate stress between organelles. However, many of these conclusions rely on indirect approaches such as ROS-sensitive fluorescent probes, pharmacological antioxidants, or global genetic deletions, which do not always discriminate between mitochondrial and extramitochondrial ROS sources or between physiological and pathological redox signals. Non-specific probes, phototoxicity during imaging, and limited temporal resolution complicate the precise quantification of mtROS dynamics and may bias interpretation toward a predominantly detrimental role of ROS, underestimating their adaptive functions.

Extramitochondrial ROS sources, primarily NADPH oxidases (NOX), represent a parallel and partly interconnected arm of redox signaling in AP. NOX1 in PACs and NOX2 in neutrophils generate superoxide anion (O_2_^-^), contributing to oxidative tissue injury and inflammatory amplification (27). From the standpoint of inter-organellar communication, the key point is that NOX-derived ROS and mtROS can reinforce one another in a feed-forward loop, thereby prolonging redox disturbances that affect multiple intracellular compartments rather than a single organelle in isolation. However, many studies have employed potent NOX inhibitors or global knockouts that affect multiple cell types, making it difficult to attribute specific effects to PAC-intrinsic NOX activity. Furthermore, the relative contribution of NOX- versus mitochondria-derived ROS likely varies across AP etiologies, disease stages, and tissue compartments. Even so, the available data support the concept that oxidative stress in AP is maintained by interconnected mitochondrial and extramitochondrial pathways.

NADPH occupies a central position at the intersection of mitochondrial metabolism, antioxidant defense, and redox signaling. By providing reducing equivalents for the glutathione system, NADPH supports regeneration of reduced glutathione (GSH) from its oxidized form (GSSG), which is essential for detoxification of hydrogen peroxide by glutathione peroxidases (28). At the same time, NADPH fuels NOX activity, illustrating how mitochondrial metabolism can indirectly influence both antioxidant protection and oxidative injury in other cellular compartments (29, 30). This dual role is particularly important for organelle crosstalk because it links mitochondrial metabolic state to redox conditions in the cytosol, peroxisomes, and membrane-associated signaling domains. However, most experimental studies have focused on static measurements of GSH/GSSG ratios or single time-point assessments of antioxidant enzyme activities, providing limited insight into the spatial and temporal dynamics of NADPH-dependent redox fluxes between mitochondria and other organelles.

Among redox-dependent regulators, HIF-1α and NRF2 exemplify how mitochondrial signals are translated into nuclear transcriptional programs that affect the broader organelle network. HIF-1α is stabilized by hypoxia or succinate accumulation and modulates transcriptional responses related to metabolism, survival, and stress adaptation. In a rat model of hyperlipidemic AP, the HIF-1α–PPARγ–mTORC1 axis promoted autophagy induction and inflammatory activation (31), whereas other studies suggested that HIF-1α may support PAC function in response to injury (32). NRF2 is a central regulator of antioxidant defense that, after dissociation from KEAP1, translocates to the nucleus and activates transcription of cytoprotective genes such as HO-1, NQO1, and GPX (33). In the context of inter-organellar communication, these transcription factors are relevant because they convert mitochondrial redox imbalance into nuclear adaptive or maladaptive responses that secondarily influence ER stress handling, autophagic capacity, and cellular survival. In AP, NRF2 activation is generally regarded as protective, although its long-term effects may differ in chronic inflammatory or neoplastic settings (34). Current AP models rarely capture this long-term dichotomy, as most experiments are terminated within hours to a few days of disease onset.

From the perspective of inter-organellar communication, mitochondrial redox signals influence not only the nucleus, but also ER stress responses, lysosomal function, and peroxisomal metabolism. Oxidative modification of ER-resident chaperones and Ca^2+^-handling proteins can aggravate the unfolded protein response and disturb Ca^2+^ release, thereby feeding back into mitochondrial dysfunction and further ROS generation. Likewise, mtROS can impair autophagic flux and lysosomal integrity, limiting the clearance of damaged mitochondria and other organelles. Peroxisomal redox buffering may also be compromised under these conditions, further weakening the cellular capacity to contain oxidative injury across organelle networks. However, in many studies, these interconnections have been inferred from parallel changes in redox markers and organellar stress readouts rather than from direct, organelle-specific manipulation of redox states. The lack of tools that can selectively modulate or monitor redox status in defined microdomains, such as MAMs, remains a major methodological limitation. Taken together, these data indicate that redox signaling is not merely a by-product of oxidative injury, but an active mechanism through which mitochondrial stress is transmitted to other organelles once damage has been initiated.

Overall, redox signaling involving mtROS, the PRX3–SRX axis, HIF-1α, NRF2, and the NOX–NADPH system constitutes a regulatory network through which mitochondrial dysfunction is communicated to the ER, nucleus, lysosomes, and peroxisomes, thereby amplifying organellar stress and shaping cell fate in AP (24, 26–34). Accordingly, mitochondrial redox signaling should be considered a central mechanism of injury propagation within the interconnected organelle system of the acinar cell.

Once calcium overload and oxidative stress have injured mitochondria, the next major determinant of outcome is whether those organelles can be removed through effective lysosomal-autophagic quality control.

Autophagy is a fundamental mechanism of cellular homeostasis that enables the removal of damaged organelles, misfolded proteins, and superfluous cytoplasmic components through lysosomal degradation. In PACs, this process is particularly important because of their high secretory load and sensitivity to metabolic stress. By eliminating dysfunctional mitochondria, autophagy, and more specifically mitophagy, is thought to help limit excessive ROS formation, release of DAMPs, and amplification of inflammatory responses in AP (35).

Selective autophagy of mitochondria (mitophagy) is triggered when damaged or depolarized mitochondria accumulate PINK1 on the outer mitochondrial membrane, leading to recruitment and activation of the E3 ubiquitin ligase Parkin. Subsequent ubiquitination of outer mitochondrial membrane proteins promotes recognition of injured mitochondria by autophagic adaptors, sequestration into autophagosomes, and lysosomal degradation. In experimental AP, activation of PINK1/Parkin-dependent mitophagy has been associated with inhibition of NLRP3 inflammasome signaling and attenuation of inflammatory activity in PACs (11). These observations support the concept that, particularly in the early phase of disease, mitophagy functions predominantly as a protective quality-control response that limits the persistence of dysfunctional mitochondria. Nevertheless, this interpretation should not be regarded as absolute, because the net effect of sustained mitophagic activity is likely to depend on disease stage, lysosomal competence, mitochondrial biogenesis, and residual cellular bioenergetic reserve.

Electron microscopy studies have demonstrated that, as early as 1–2 hours after AP induction, numerous autophagosomes and autophagolysosomes containing mitochondrial remnants appear in PACs, indicating rapid activation of mitophagy (10). When this process remains functionally coupled to lysosomal clearance, morphological evidence of mitochondrial injury decreases over time and cellular architecture partially normalizes, consistent with partial restoration of intracellular homeostasis (10). By contrast, in severe AP, mitophagy appears insufficient or defective, with accumulation of damaged mitochondria and enlarged autophagic vacuoles, persistent ROS generation, enhanced DAMP release, and intensification of apoptosis and necrosis. Blockade of autophagic flux is suggested by persistence of autophagosomes, accumulation of p62-positive structures, and defective autophagosome–lysosome fusion (36). These findings indicate that defective mitophagy contributes primarily to the persistence and amplification of mitochondrial injury rather than representing the initiating pathogenic event.

Available data also suggest stage-dependent effects. Early mitophagy appears adaptive because it removes damaged mitochondria before they amplify oxidative and inflammatory injury, whereas later in the disease course the same pathway may become less effective, or even functionally disadvantageous, in the setting of profound ATP depletion, lysosomal dysfunction, and impaired mitochondrial replenishment. Under such conditions, continued sequestration of mitochondria into incompletely resolved autophagic vacuoles may further reduce the already limited pool of functional organelles and thereby contribute to energetic collapse rather than recovery. However, this possibility remains inferential, because most studies rely on single or few time points and on qualitative ultrastructural assessment, which cannot fully capture the dynamic nature of mitophagic flux or exclude cell-type–specific differences within the pancreatic microenvironment.

The protective role of mitophagy in AP is further supported by findings that upregulation of mitophagy-related proteins (such as PINK1, Parkin, and other mitophagy receptors) is associated with more efficient clearance of depolarized mitochondria, reduced release of pro-apoptotic factors, and attenuation of acinar cell death (11, 35). Conversely, genetic or pharmacological impairment of mitophagy is associated with accumulation of dysfunctional mitochondria, energetic failure, and progression of local and systemic inflammation (35). At the same time, many of these interventions rely on global knockouts or systemic modulators of autophagy, making it difficult to distinguish PAC-intrinsic effects from changes in immune cells, endothelial cells, or other pancreatic cell populations. This limitation is important when interpreting whether mitophagy is uniformly beneficial in PACs, because whole-body manipulation may alter inflammatory signaling, immune-cell recruitment, vascular responses, or systemic metabolism, each of which can secondarily influence mitochondrial stress in the pancreas. Therefore, conclusions regarding PAC-specific mitophagy should be considered provisional unless confirmed in cell-restricted and temporally controlled models. In addition, commonly used pharmacological tools to “enhance” or “inhibit” autophagy, including chloroquine, bafilomycin A1, and rapamycin, exert pleiotropic effects beyond the autophagy–lysosome system, further complicating mechanistic interpretation in AP models.

The final stage of autophagy – lysosomal degradation of autophagosomal cargo – is a critical bottleneck in AP. In many experimental models, this stage is disrupted: lysosomal enzymes are prematurely activated or mislocalized, lysosomal membrane integrity is compromised, and lysosome–autophagosome fusion is impaired, all of which lead to accumulation of large autophagic vacuoles and impaired PAC function (37). These observations indicate that AP is characterized not simply by excessive or insufficient autophagy, but by a qualitative defect in autophagic flux centered largely at the lysosomal level. Such defects are expected to aggravate pre-existing mitochondrial injury by preventing effective clearance of damaged organelles. However, the temporal position of lysosomal dysfunction within AP pathogenesis remains incompletely resolved. It may emerge as a contributing factor to defective organelle turnover, as a downstream consequence of broader intracellular injury, or both, depending on disease stage and model context. Moreover, most available studies focus on the early phase of pancreatitis, whereas the behavior of the autophagy–lysosome pathway during later stages marked by necrosis, infection, or tissue regeneration remains far less well defined.

Thus, functional crosstalk between mitochondria and lysosomes via autophagy and mitophagy represents a double-edged mechanism: it can protect PACs by removing damaged organelles but, when dysregulated, is associated with accumulation of dysfunctional mitochondria, amplification of ROS and DAMP signaling, and a shift from adaptive stress responses to irreversible cell death (10, 11, 35–37). Accordingly, mitophagy in AP should be interpreted not as an invariably protective pathway, but as a context-dependent response whose net effect likely depends on disease stage, residual cellular bioenergetic reserve, lysosomal competence, and the capacity for mitochondrial replenishment. Taken together, available evidence indicates that mitophagy is primarily a secondary protective response to mitochondrial injury, whereas impaired mitophagy and lysosomal dysfunction contribute to progression and amplification of acinar cell damage in AP. Although experimental models clearly establish the importance of the mitochondria–lysosome axis in AP pathogenesis, variability in AP induction protocols, limitations of current autophagy and mitophagy assays, and the lack of successful clinical translation of autophagy-targeted interventions underscore the need for more refined, pancreas-specific, and temporally resolved approaches before this system can be exploited therapeutically in patients.

Mitochondrial quality control is determined not only by lysosomal competence but also by the cytoskeletal architecture that spatially organizes mitochondria, autophagosomes, and lysosomes within PACs. Disturbance of this architecture may alter mitochondrial positioning, promote abnormal mitochondrial remodeling, and impair their functional integration with trafficking pathways required for effective organelle surveillance and turnover. Mitochondrial dynamics are a complex, tightly regulated process that depend on continuous interaction with the cytoskeleton to maintain appropriate organelle positioning, morphology, and connectivity within the mitochondrial network. In PACs, efficient distribution of mitochondria toward regions of high ATP demand, such as perigranular and subplasmalemmal domains, is essential for sustaining exocytosis, ion transport, and Ca^2+^ buffering.

Mitochondrial movement along microtubules is mediated by motor proteins of the kinesin and dynein families, which transport mitochondria to and from specific cellular regions, thereby matching local energy supply with demand. In PACs, such targeted trafficking is necessary to position mitochondria near zymogen granules, perinuclear areas, and the PM, thereby supporting stimulus–secretion coupling and Ca^2+^ homeostasis. Experimental disruption of microtubules using pharmacological agents results in impaired mitochondrial motility, perinuclear clustering, and reduced delivery of mitochondria to high-demand sites, culminating in energetic insufficiency and disturbances in Ca^2+^ signaling (37). However, microtubule inhibitors are highly non-specific and affect many additional processes, including vesicular trafficking, mitosis, and immune cell functions. Consequently, it is difficult to attribute the protective or deleterious effects observed in AP models solely to altered mitochondrial motility, and the possibility of off-target actions must be carefully considered when interpreting these data.

Key proteins that regulate mitochondrial dynamics include DRP1 (dynamin-related protein 1), which orchestrates mitochondrial fission; mitofusins (MFN1 and MFN2), which mediate outer-membrane fusion; and OPA1 (optic atrophy 1), which controls inner-membrane fusion and cristae structure. These proteins interact directly or indirectly with cytoskeletal elements, thereby coupling morphological remodeling of the mitochondrial network to changes in cell shape, polarity, and motility. In AP models, excessive activation of DRP1 and/or downregulation of MFN1/2 and OPA1 has been associated with pathological mitochondrial fragmentation, loss of ΔΨm, decreased ATP production, and accumulation of dysfunctional mitochondria (35, 38). However, the role of fission is not uniformly detrimental; prompt segregation and removal of damaged mitochondrial segments may be protective under moderate stress, whereas broad pharmacological inhibition of DRP1 (e.g., with Mdivi-1) can interfere with physiological quality control. Moreover, many DRP1 inhibitors have important off-target effects on other GTPases and cellular pathways, so conclusions about the benefits of “DRP1 blockade” in AP should be treated with caution.

Under pathophysiological conditions, particularly during necroptosis and other regulated forms of cell death, cytoskeletal remodeling and mitochondrial dynamics become tightly intertwined. Pro-inflammatory signaling and Ca^2+^ overload can activate kinases that phosphorylate DRP1 and other fission-related proteins, thereby promoting mitochondrial fragmentation, ΔΨm loss, and increased ROS generation (39). Simultaneously, actin cytoskeleton rearrangements influence mitochondrial constriction sites, contact with the ER, and positioning near lysosomes, collectively shaping the likelihood that damaged mitochondria will undergo mitophagy versus progressing toward permeabilization and DAMP release.

The interaction between mitochondria and the cytoskeleton also influences the spatial organization of other key processes in AP, including autophagosome trafficking, lysosomal fusion, and immune cell recruitment to the injured pancreas. Disruption of microtubule- or actin-dependent mitochondrial positioning can misalign zones of Ca^2+^ entry, ROS production, and ATP generation, thereby promoting local energy crises and facilitating progression from adaptive stress responses to necrosis. However, from a translational standpoint, targeting cytoskeletal–mitochondrial interactions is challenging because systemic microtubule modulators or actin-targeting drugs have narrow therapeutic windows and substantial toxicity, particularly for proliferating cells such as bone marrow progenitors and intestinal epithelium. Furthermore, patients with AP often receive medications (e.g., sedatives, antibiotics, and vasoactive agents) that can influence cytoskeletal organization and mitochondrial function, complicating the clinical applicability of strategies derived from reductionist models.

Taken together, available data support a central role of mitochondria–cytoskeleton crosstalk in maintaining mitochondrial distribution, morphology, and quality control in PACs and in determining whether cells adapt to injury or progress toward death in AP (35, 37–39).

### 3.3 Mitochondria and the plasma membrane

At the cell periphery, these intracellular mechanisms converge on the plasma membrane, where calcium entry both drives physiological secretion and, when excessive, perpetuates mitochondrial injury. Mitochondria closely interact with the plasma membrane (PM) to regulate intracellular calcium homeostasis and local energy supply. In PACs, physiological stimulation induces Ca^2+^ entry through the PM via store-operated Ca^2+^ entry (SOCE), where Orai1 channels are activated by STIM1 in response to depletion of ER Ca^2+^ stores. Mechanosensitive Piezo1 channels can also contribute under specific conditions (40). The resulting Ca^2+^ signal is rapidly taken up by mitochondria via the MCU, which buffers local Ca^2+^ concentrations, stimulates oxidative metabolism, and helps preserve cell viability.

Under physiological conditions, mitochondria localized near the apical membrane form a buffering zone that restricts the spread of Ca^2+^ signals from the subplasmalemmal region to the cell interior, thereby protecting PACs from Ca^2+^-induced injury (16). Ca^2+^ microdomains arising at close contact between the PM and mitochondria enable rapid mitochondrial Ca^2+^ uptake, activation of TCA cycle enzymes, and stimulation of ATP production, a critical energy source for enzyme secretion and maintenance of electrolyte gradients (41). At the same time, direct analysis of these microdomains remains technically difficult. Conventional Ca^2+^ indicators average signals over relatively large cytosolic volumes, whereas genetically encoded Ca^2+^ sensors targeted to defined subcellular compartments are only beginning to be applied systematically in pancreatic models. Consequently, the spatial and temporal organization of Ca^2+^ signaling at mitochondria–PM interfaces is still inferred in many studies rather than directly visualized. Moreover, many observations derive from isolated acinar cells or tissue slices, in which extracellular matrix architecture, membrane tension, and mechanosensitive channel behavior may differ substantially from the *in vivo* pancreas.

In pathological states such as AP, excessive Ca^2+^ influx through Orai1 or mechanosensitive Piezo1 channels can overwhelm mitochondrial buffering capacity and promote mitochondrial dysfunction (42). Reduced ATP levels compromise the activity of SERCA and PMCA, causing cytosolic Ca^2+^ retention and exacerbating Ca^2+^ imbalance (43). Thus, mitochondria not only buffer Ca^2+^ but also indirectly modulate the functional activity of PM Ca^2+^ channels. Once their buffering capacity is exceeded, sustained elevation of subplasmalemmal Ca^2+^ favors prolonged SOCE activation and a self-reinforcing injury loop (17). In this context, the PM–mitochondria interface should be viewed less as an isolated source of injury than as a site at which disturbed ion entry, impaired ATP supply, and defective Ca^2+^ clearance reinforce one another. However, the magnitude and kinetics of this loop are likely to vary according to AP etiology, local mechanical conditions such as edema or fat infiltration, and systemic modifiers including acidosis and hypoxia, variables that are only partly reproduced in standard experimental models. In addition, genetic manipulation of MCU or Orai1 has often been performed in global rather than PAC-specific settings, making it difficult to distinguish direct acinar-cell protection from systemic immune, vascular, or metabolic effects that may secondarily influence pancreatitis severity.

Thus, mitochondria play a central role in Ca^2+^ buffering, energetic responses to Ca^2+^ signaling, and the regulation of PM Ca^2+^ channel activity at PAC borders. Experimental data convincingly demonstrate that disruption of this bidirectional interaction in AP creates a vicious circle of Ca^2+^ toxicity, energy depletion, and progressive cell death (16, 17, 40–43). Accordingly, this interface is best interpreted as a dynamic regulatory platform in which mitochondrial performance determines whether PM Ca^2+^ entry remains physiologically coupled to secretion or shifts toward sustained injury signaling.

### 3.4 Mitochondria and the nucleus

When mitochondrial depolarization, redox imbalance, and calcium dysregulation persist, the consequences are no longer limited to local bioenergetic failure and begin to reprogram nuclear stress responses. Beyond their role in ATP production, mitochondria function as stress sensors that transmit retrograde signals to the nucleus, thereby reshaping gene-expression programs and determining whether a cell adapts or undergoes death. Fluctuations in ΔΨm, changes in matrix Ca^2+^, alterations in the NAD^+^/NADH ratio, and increased mtROS modulate nuclear transcription factors and chromatin regulators, while the release of mitochondrial DAMPs, including mtDNA and cardiolipin, links mitochondrial injury to inflammatory signaling and sterile immune activation.

In AP, mitochondrial dysfunction, including ATP depletion, mtROS overproduction, and loss of ΔΨm, promotes nuclear activation of stress-responsive transcriptional programs that enhance cytokine and chemokine expression and thereby contribute to pancreatic inflammation (44). Mitochondrial DAMPs released from injured or necrotic acinar cells can further amplify this response by activating DNA-sensing pathways and perpetuating sterile inflammation in the pancreas (44, 45). Thus, mitochondria-to-nucleus signaling in AP extends beyond metabolic failure and directly influences inflammatory gene expression.

ATF4 is a key effector of the integrated stress response that connects mitochondrial stress, ER stress, and redox imbalance to nuclear transcriptional reprogramming. In AP, increased ATF4 expression has been associated with induction of stress-related transcriptional programs, altered autophagy, and progression of acinar injury (46, 47). At the same time, ATF4-dependent signaling may support adaptive autophagy under moderate stress, indicating that its effects depend on the severity and duration of mitochondrial dysfunction rather than representing a purely detrimental pathway (26).

Transcription factor p53 is another regulator through which mitochondrial stress is translated into nuclear responses. Under conditions of severe oxidative or genotoxic stress, p53 promotes transcription of pro-apoptotic genes such as Bax and PUMA, thereby facilitating mitochondrial outer membrane permeabilization and apoptosis (48, 49). In the setting of AP, this pathway is relevant primarily because it links mitochondrial damage to nuclear decisions on cell fate and may influence the balance between controlled cell elimination and lytic injury. In AP models, p53 activation has been associated with both apoptotic containment of damaged cells and aggravation of necrotic inflammation, depending on injury severity (48, 49).

NRF2 is a master regulator of antioxidant and cytoprotective gene expression that responds to alterations in mitochondrial redox state. When mitochondrial and cytosolic ROS levels rise, NRF2 is released from KEAP1, accumulates in the nucleus, and induces a transcriptional program that supports antioxidant defense and cellular recovery (52). In AP models, NRF2 activation generally reduces acinar cell injury, stabilizes mitochondrial function, limits lipid peroxidation, and attenuates inflammatory responses (52, 53). Genetic NRF2 deficiency or pharmacological inhibition typically aggravates disease severity, supporting the concept that NRF2-mediated transcription is an important adaptive component of mitochondria–nucleus communication in AP (54, 55). Because the present focus is acute organelle crosstalk, the broader oncogenic implications of chronic NRF2 activation are beyond the scope of this section.

HIF-1α links mitochondrial metabolism and oxygen consumption to nuclear programs involved in metabolic adaptation to stress. In AP, particularly in severe forms associated with microcirculatory impairment, HIF-1α stabilization may support glycolytic adaptation but can also accompany inflammatory activation and pathological autophagy (31). This duality again emphasizes that mitochondria-to-nucleus signaling is context-dependent: transient activation may be adaptive, whereas sustained activation may reinforce injury.

Taken together, mitochondria and the nucleus form a tightly coupled signaling axis in which mitochondrial dysfunction in AP activates nuclear regulators such as NF-κB, ATF4, p53, NRF2, and HIF-1α, thereby influencing inflammation, autophagy, antioxidant defense, and cell-death pathways (8, 26, 31, 44–55). Conversely, these nuclear responses feedback on mitochondrial quality control and stress tolerance, making the mitochondria–nucleus axis a central component of inter-organellar communication in acinar cells.

### 3.5 Mitochondria and lipid droplets

Metabolic adaptation to this stress also depends on how mitochondria interface with lipid-handling organelles. Under physiological conditions, LDs and mitochondria form a dynamic functional interaction that is essential for the regulation of energy metabolism, lipid turnover, and maintenance of cellular homeostasis. LDs are not merely depots of neutral lipids (triacylglycerols and cholesteryl esters), but active participants in lipogenesis, lipolysis, and detoxification of lipid excess (56, 57). LD biogenesis begins in the ER, where accumulation of neutral lipids within the membrane gives rise to lens-like structures that subsequently bud into the cytoplasm and acquire a phospholipid monolayer with associated proteins (56).

Mitochondria adjacent to LDs, so-called peridroplet mitochondria (PDMs), display distinct functional properties, including increased ATP synthesis and a specific proteome that favors the energy-efficient formation of new LDs and protects the cell from lipotoxicity (58). LDs also exert antioxidant functions by buffering excess free FAs and preventing excessive ROS formation, which can damage the mitochondria and other organelles (57, 58). This mitochondria–LD axis is of particular importance in hypertriglyceridemia-induced AP (HTG-AP), a subtype whose clinical relevance has increased in recent years and which is associated with greater severity and recurrence risk in contemporary analyses (59, 60). In HTG-AP, hydrolysis of triglyceride-rich lipoproteins and peripancreatic fat generates excessive amounts of non-esterified fatty acids (NEFAs), particularly unsaturated long-chain fatty acids, which exceed the buffering capacity of LDs and promote mitochondrial depolarization, Ca^2+^ dysregulation, oxidative stress, and necrotic cell death (61).

In AP, LD–mitochondria interactions undergo profound alterations as part of the pathogenic mechanism of PAC injury. Recent studies have shown that in induced AP, particularly of alcoholic origin, there is a marked accumulation of LDs in the pancreatic cell cytoplasm, accompanied by mitochondrial dysfunction and activation of inflammatory and autophagic processes (62, 63). Using the fluorescent probe MLD-1, investigators have demonstrated that in pancreatic inflammation, LD levels increase significantly and correlate with impaired mitochondrial function, confirming a close functional link between disturbed energy metabolism and lipid imbalance in AP (63). However, MLD-1 provides only semi-quantitative information on neutral lipid content and does not distinguish between triglycerides, cholesteryl esters, or potentially more toxic lipid species. Moreover, most measurements are performed at a few fixed time points in small animal cohorts. As a result, these data support a correlation between LD accumulation and mitochondrial dysfunction, but do not establish whether altered LD–mitochondria contacts are causative drivers of injury or secondary markers of broader metabolic collapse.

Electron microscopy in alcohol-induced pancreatitis in alcohol dehydrogenase-deficient mice revealed pronounced mitochondrial swelling and fragmentation, together with excessive LD accumulation in PACs. Concurrently, elevated levels of triglycerides, FAs, and fatty acid ethyl esters were detected in pancreatic tissue, indicating active involvement of LDs in pathological lipid metabolism (64). This alcohol dehydrogenase-deficient model emphasizes the contribution of non-oxidative ethanol metabolites (fatty acid ethyl esters) to lipotoxic injury, but represents a very specific experimental scenario that may not fully recapitulate biliary, hypertriglyceridemic, or drug-induced AP. Furthermore, EM provides high spatial resolution, but only static snapshots of LD–mitochondria proximity and tissue processing can introduce artifacts in LD size and distribution. Quantitative ultrastructural analyses are rarely linked to functional assays of β-oxidation, lipolysis, or mitochondrial respiration in the same cells, which limits integrated interpretation of structure–function relationships.

In HTG-AP, lipotoxicity is unlikely to be explained by mitochondrial overload alone. Peroxisomes contribute to the initial β-oxidation of very-long-chain and structurally complex fatty acids, thereby reducing the burden transferred to mitochondria; when this auxiliary pathway is insufficient, incompletely processed lipid species and reactive oxygen metabolites may further aggravate mitochondrial dysfunction. Although direct evidence in PACs remains limited, this concept is biologically consistent with current mechanistic models of HTG-AP, in which excessive fatty acid flux and oxidative injury are central determinants of disease severity (60, 61).

In addition, alcohol activates the AMP-activated protein kinase (AMPK) signaling pathway, which disrupts autophagic flux and aggravates lysosomal and mitochondrial dysfunction, whereas LD accumulation likely represents a compensatory response to cellular stress (62). Whether LD expansion in this context is predominantly adaptive (sequestering toxic FAs and fatty acid ethyl esters away from the mitochondria) or maladaptive (sustaining a pool of lipotoxic precursors and interfering with organelle quality control) remains unresolved and may depend on the timing, severity, and etiology of AP.

Thus, in hypertriglyceridemic disease, LDs should be viewed not only as lipid storage organelles but also as transient buffering compartments within a broader mitochondria–peroxisome–LD network. Once the combined oxidative capacity of this system is exceeded, LD expansion becomes insufficiently protective and may convert into a feed-forward mechanism sustaining NEFA accumulation, ROS generation, bioenergetic collapse, and necroinflammation (59, 61).

### 3.6 Mitochondria and secretory granules

Because pancreatic injury is ultimately expressed through disturbed secretion and premature enzyme activation, the consequences of mitochondrial dysfunction must also be considered within the apical secretory domain. Pancreatic acinar cells exhibit exceptionally high secretory activity and therefore depend on close spatial and functional coupling between mitochondria and the apical region enriched in zymogen granules (ZGs). Mitochondria represent the dominant ATP source in PACs and are strategically positioned around granule-rich domains, where they respond to local Ca^2+^ elevations and provide energy for exocytosis (17). Perigranular mitochondrial clusters form local Ca^2+^ buffers that limit the spread of Ca^2+^ signals and support apical–basal polarity during stimulus–secretion coupling.

During physiological stimulation by ACh or CCK, repetitive Ca^2+^ spikes in the apical cytosol trigger both zymogen granule exocytosis and mitochondrial activation, ensuring that ATP generation is tightly coupled to the energetic cost of enzyme secretion (66). Mitochondria located in close apposition to exocytotic sites may therefore be regarded as metabolic integrators that coordinate local Ca^2+^ signaling with ATP supply.

However, the available evidence does not yet establish a specific, direct mitochondria–zymogen granule communication system in PACs. Current data more convincingly support spatial proximity and functional interdependence within the apical secretory domain than a dedicated organelle contact site or a validated bidirectional signaling axis. Accordingly, the relationship between mitochondria and ZGs is more accurately interpreted as a biologically plausible functional association rather than a definitively proven direct interaction.

In healthy PACs, ZGs store inactive digestive proenzymes, such as trypsinogen, and maintain strict spatial and biochemical segregation from lysosomal hydrolases, while nearby mitochondria help sustain the energetic requirements of regulated secretion. Under pathological conditions, disruption of this apical microenvironment is likely to contribute to abnormal secretion and intracellular zymogen activation, but the specific contribution of mitochondria to these events appears to be predominantly indirect. Experimental studies have shown that trypsinogen activation can occur within secretory granules independently of canonical fusion with lysosomes. Even in the absence of Rab7, a small GTPase involved in late endosomal and lysosomal trafficking, intracellular trypsinogen activation and pancreatic injury persist, suggesting that active cathepsin B can localize within ZGs (67). Another study confirmed Rab7 presence on ZG membranes and its role in granule maturation while reporting no major effect on stimulus-induced exocytosis or bulk autophagy; Rab7 deficiency led to an increased number of small ZGs, favoring excessive accumulation of digestive enzymes and creating conditions permissive for autoactivation and cellular injury (68).

Importantly, these findings primarily inform granule biology and vesicular trafficking rather than direct mitochondria–ZG signaling. Most available models manipulate pathways, such as Rab7-dependent trafficking, that affect multiple vesicular compartments simultaneously, including late endosomes and lysosomes. Consequently, the resulting phenotypes cannot be attributed specifically to altered mitochondria–ZG communication.

From a functional standpoint, perigranular mitochondria are important for providing ATP to support exocytosis and for buffering local Ca^2+^ elevations, which under physiological conditions helps preserve acinar polarity and secretory control (17, 65, 66). In AP, once mitochondrial Ca^2+^ handling is overwhelmed and ΔΨm collapses, mitochondrial dysfunction may secondarily promote a secretory environment favorable to premature enzyme activation through ATP depletion, defective local Ca^2+^ buffering, oxidative stress, and impaired vesicular homeostasis. However, direct experimental evidence showing that selective alteration of mitochondrial positioning or signaling specifically at ZG-rich domains is sufficient to trigger premature trypsinogen activation remains lacking. Thus, the causal role of mitochondria–granule proximity in AP should be considered suggestive rather than established.

Overall, the available data support a cautious model in which mitochondria in the apical domain of PACs function as local ATP suppliers and Ca^2+^ buffers that help maintain the secretory apparatus under physiological conditions, whereas mitochondrial failure in AP may indirectly destabilize the granule-rich apical region and facilitate pathological zymogen activation. At present, this concept is best framed as a hypothesis-generating interpretation supported mainly by spatial and functional evidence, rather than as a conclusively demonstrated direct organelle interaction.

### 3.7 Mitochondria and autophagosomes

Efficient mitophagy depends not only on lysosomal degradation but also on upstream cargo sequestration and vesicle maturation. In AP, interactions between mitochondria and autophagosomes occur mainly during mitophagy, a selective form of autophagy that removes damaged mitochondria and thereby contributes to intracellular quality control. Because the core PINK1–Parkin-dependent mechanism of mitophagy has been outlined above, this section focuses primarily on regulators that influence autophagosome formation, cargo handling, and mitophagic efficiency in PACs. Mitophagy is activated in response to loss of ΔΨm and ROS accumulation and is therefore positioned at the intersection of mitochondrial injury, organelle quality control, and inflammatory signaling.

Another mechanism of interest is VMP1-dependent mitophagy, which is activated early in pancreatitis. VMP1 interacts with components of the mitochondrial quality-control machinery and regulates mitochondrial fragmentation and subsequent incorporation of damaged organelles into autophagosomes (35). Reduced VMP1 expression has been linked to defective mitophagy, underscoring its importance in the cellular protective response (35). However, VMP1 is a broadly acting autophagy-related protein, and most available evidence is derived from global genetic manipulations or systemic interventions that affect multiple forms of autophagy rather than mitophagy alone. This makes it difficult to determine to what extent the beneficial effects observed in AP models reflect specifically improved mitochondrial quality control, as opposed to broader changes in autophagosome biogenesis, ER stress adaptation, or secretory pathway homeostasis. In addition, the temporal window during which VMP1-dependent mitophagy remains beneficial is still incompletely defined. Its activation may be advantageous in early disease, whereas in advanced pancreatitis marked by lysosomal insufficiency and impaired mitochondrial replenishment, continued sequestration of mitochondria into incompletely resolved autophagic vacuoles could theoretically aggravate bioenergetic decline. At present, however, this possibility remains mechanistically plausible rather than directly demonstrated in PACs.

Not all autophagic pathways are equally effective. Overexpression of the GTPase Rab9 in the pancreas shifts autophagosome formation from the canonical LC3-II-dependent pathway to a non-canonical Rab9-dependent route, which appears to be less efficient at clearing defective mitochondria. This has been associated with accumulation of damaged organelles, augmented inflammation, and worse AP outcomes (70). Moreover, the protein MRG15 (MORF4L1), whose expression is elevated in hyperlipidemic pancreatitis, inhibits mitophagy by promoting degradation of TUFM, a mitochondrial translation factor; this is associated with enhanced PAC apoptosis and more severe disease (71). These findings indicate that the route of autophagosome biogenesis and the integrity of mitochondrial translational machinery both critically influence the efficiency of mitophagic clearance. Thus, protection in AP appears to depend not merely on the presence of mitophagy, but on whether the process is timely, selective, and completed successfully. Efficient flux may be beneficial during the early adaptive phase, whereas incomplete or dysregulated mitophagy in advanced disease may fail to eliminate damaged organelles and may instead intensify organelle loss without restoring mitochondrial function.

The DAMP molecule CIRP (Cold-inducible RNA-binding protein) also plays a distinct role in AP pathogenesis. Excess CIRP released from necrotic cells has been reported to be associated with mitochondrial dysfunction, impaired autophagy, and pyroptosis (inflammatory programmed cell death) in PACs. Genetic or pharmacological blockade of CIRP has been associated with restoration of mitochondrial function, reduction of ROS generation, and suppression of pyroptosis, highlighting its potential as a therapeutic target (72). Nevertheless, these findings should be interpreted cautiously, because improvement after systemic blockade of an upstream inflammatory mediator does not necessarily prove a direct, PAC-specific effect on mitophagy itself. Rather, such benefit may reflect broader attenuation of inflammatory stress, which secondarily preserves mitochondrial integrity and autophagic function.

Taken together, the available data indicate that mitophagy is a crucial component of the cellular defense program in AP, and pathways involving Sestrin2, VMP1, Rab9, MRG15, and CIRP collectively determine whether damaged mitochondria are efficiently removed or instead accumulate and fuel inflammatory and cell-death cascades (35, 69–72). Overall, current evidence supports a predominantly protective role of mitophagy in the early phase of AP, but it does not exclude stage-dependent effects in which prolonged or ineffective mitochondrial clearance could become maladaptive under conditions of severe ATP depletion, defective lysosomal processing, and limited mitochondrial biogenesis. However, much of the available evidence remains associative, and the temporal sequence linking mitochondrial injury, autophagosome formation, and downstream inflammatory amplification has not yet been fully resolved in PACs. Further progress will require inducible, pancreas-specific, and time-resolved experimental models capable of distinguishing PAC-intrinsic mitophagy defects from systemic or multicellular consequences of global genetic manipulation.

### 3.8 Mitochondria and peroxisomes

A parallel metabolic buffering system is provided by peroxisomes, which become especially important when lipid overload exceeds mitochondrial oxidative capacity. The mitochondria–peroxisome crosstalk in AP therefore involves intertwined mechanisms of antioxidant defense, inflammatory control, and energy metabolism. Mitochondrial injury in AP is accompanied by decreased ΔΨm, elevated ROS levels, activation of the NLRP3 inflammasome, and pyroptosis, ultimately leading to PAC necrosis (73). Mitochondria and peroxisomes cooperate in fatty-acid metabolism and maintenance of cellular redox balance, with peroxisomes preferentially oxidizing very-long-chain and branched-chain fatty acids that are subsequently processed further by mitochondria.

This interaction is likely to be especially important in HTG-AP, in which overwhelming delivery of triglyceride-derived non-esterified fatty acids (NEFAs) creates a lipotoxic environment that exceeds the oxidative capacity of a single organelle system. Peroxisomes are uniquely suited to initiate the shortening of very-long-chain and structurally complex fatty acids that are inefficient substrates for mitochondria, whereas mitochondria complete downstream oxidation and ATP-linked energy production; disruption of this division of labor may therefore intensify both lipid accumulation and oxidative injury (75, 76). Recent mechanistic evidence in HTG-AP further supports this concept by showing that lipolysis of circulating triglycerides generates injurious NEFAs that correlate with disease severity and organ failure, thereby providing a clinically relevant context in which insufficient mitochondrial–peroxisomal fatty acid handling may become pathogenic (61).

The UCP2/SIRT3/PGC-1α signaling axis, activated by matrine, plays an important role in limiting oxidative damage by decreasing mtROS production, inhibiting ferroptosis, and preserving mitochondrial function while also implicating peroxisomal metabolism in redox homeostasis (77). Similarly, resveratrol activates the SIRT1/PGC-1α axis, enhancing mitochondrial biogenesis, reducing NLRP3 inflammasome activation, and improving microcirculation – effects that are critical in severe AP (73). These pathways likely influence peroxisomal function indirectly, particularly in the context of FA β-oxidation and antioxidant enzyme expression, but most available data rely on systemic administration of matrine or resveratrol and on whole-pancreas or serum readouts. As a result, it remains uncertain to what extent the observed protection reflects direct normalization of mitochondria–peroxisome interactions in acinar cells versus broader vascular, immune, or hepatic effects. In addition, compounds such as resveratrol exhibit pleiotropic actions (on sirtuins, ion channels, endothelial function, and platelet activity), which complicates attribution of their benefits to a specific mitochondrial–peroxisomal axis.

From a mechanistic perspective, peroxisomal β-oxidation dysfunction in AP would be expected to exert a dual detrimental effect: first, by impairing the shortening of fatty acids before their transfer to mitochondria, and second, by weakening peroxisomal control of hydrogen peroxide and related redox signals. Under lipotoxic conditions, excessive peroxisomal substrate flux may itself become a source of oxidant stress, while inadequate catalase-dependent detoxification can facilitate ROS spillover toward mitochondria, thereby aggravating membrane depolarization, inflammasome signaling, and bioenergetic collapse (75, 76). Although direct quantification of peroxisomal β-oxidation defects in PACs is still lacking, the available metabolic framework strongly suggests that failure of this buffering pathway may be one of the underrecognized amplifiers of lipotoxic injury in HTG-AP rather than a merely secondary epiphenomenon.

PPAR-γ is particularly important in regulating the activity of the antioxidant enzymes catalase and HO-1. In PACs stimulated with cerulein and resistin, pro-inflammatory processes are activated, but α-lipoic acid, via PPAR-γ activation, increases HO-1 and catalase expression, suppresses ROS and IL-6 levels, and thereby indicates functional integration of peroxisomal and mitochondrial antioxidant defenses (78). In addition, PPAR-γ activation inhibits NF-κB–dependent activation of alveolar macrophages, reducing systemic inflammation driven by exosomes released from damaged pancreatic cells. Emodin modulates this pathway, illustrating an indirect link between peroxisome–mitochondria signaling and inter-organ communication in AP (79). Yet these PPAR-γ–centered studies again employed systemic pharmacological agonists with wide-ranging effects on adipose tissue, liver, and immune cells, and they rarely included detailed analyses of peroxisomal number, morphology, or enzyme activity in the pancreas. Thus, although they convincingly demonstrate modulation of antioxidant and inflammatory pathways, the precise contribution of peroxisome-specific changes versus global metabolic reprogramming remains to be clarified.

This limitation is particularly relevant when extrapolating these findings to HTG-AP, because hyperlipidemic disease states impose continuous fatty acid excess and may shift the mitochondria–peroxisome relationship from adaptive cooperation to maladaptive overload. In such conditions, insufficient peroxisomal fatty acid processing could increase mitochondrial substrate pressure, whereas persistent mitochondrial dysfunction would in turn impair completion of oxidative disposal, establishing a self-reinforcing loop of NEFA accumulation, ROS production, and necroinflammation (60, 61).

Taken together, the available data support the concept that cooperation between mitochondria and peroxisomes is critical for maintaining metabolic and redox equilibrium in AP; disruption of this axis promotes energetic depletion, oxidative stress, inflammasome activation, and progression of cellular injury (73, 74, 77–79). In addition, emerging evidence indicates that this axis deserves particular attention in HTG-AP, where peroxisomal β-oxidation dysfunction may substantially magnify lipotoxic stress by limiting safe fatty acid processing upstream of mitochondria. Future studies should therefore move beyond indirect pharmacological inference and directly assess peroxisomal abundance, catalase activity, β-oxidation flux, and mitochondria–peroxisome contact remodeling in PACs under hypertriglyceridemic conditions.

### 3.9 Summary

**Figure 3:**
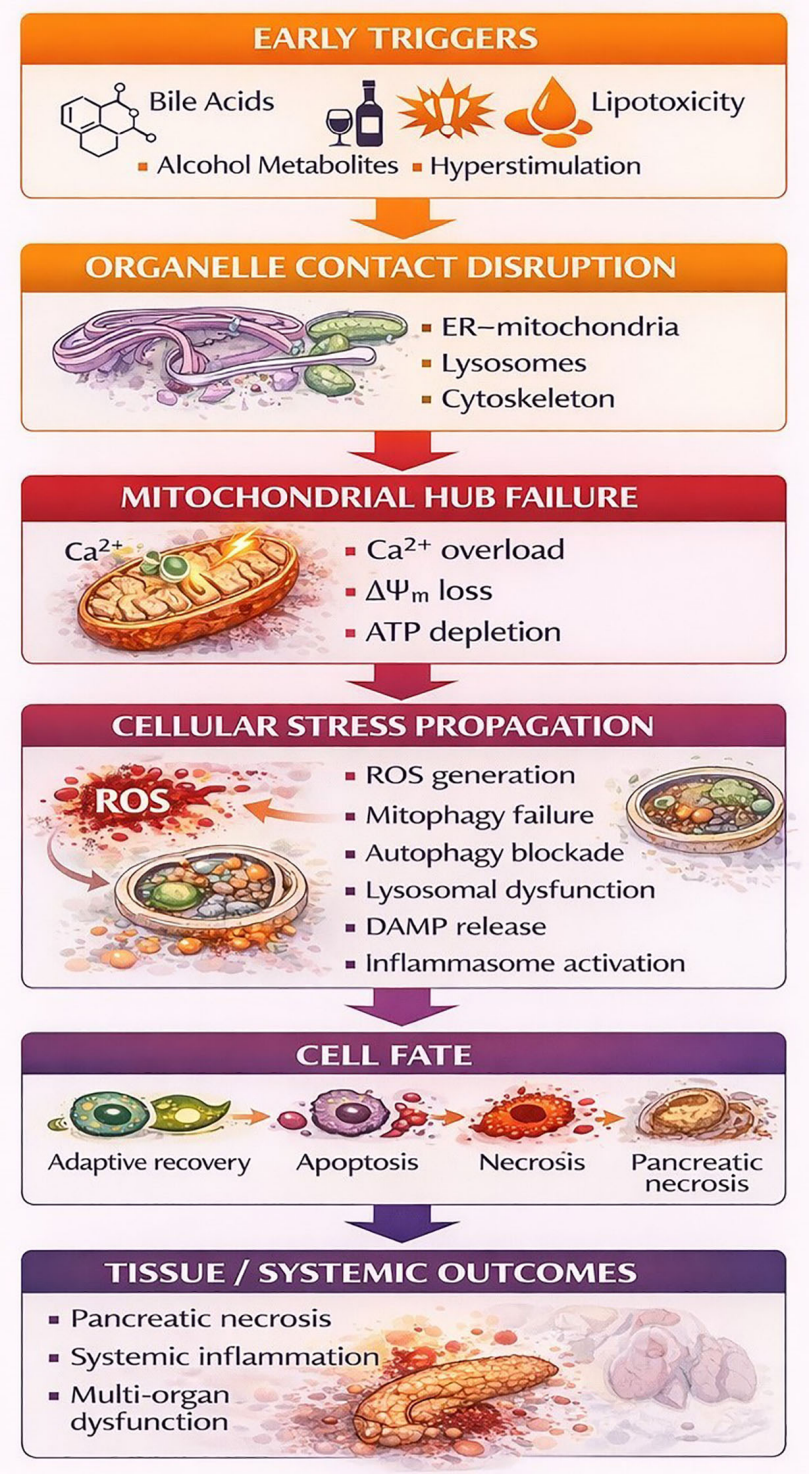
Integrative mitochondrial hub framework in acute pancreatitis.

Taken together, the available experimental evidence supports a hierarchical model of AP pathogenesis centered on the mitochondrial hub (**Figure 3**), in which early pathogenic triggers, including toxic calcium signals, bile acid exposure, alcohol metabolites, hyperstimulation, and lipotoxic stress, initiate progressive remodeling of mitochondrial contact sites with the ER, lysosomes, the cytoskeleton, and other organelles. The sequence begins with ER–mitochondria calcium maladaptation, continues through redox amplification and lysosomal-autophagic failure, and then extends to the cytoskeletal, plasma-membrane, nuclear, lipid, peroxisomal, and secretory consequences of bioenergetic collapse. As these contact interfaces become structurally and functionally disturbed, organelle crosstalk loses its spatial precision and regulatory efficiency, resulting in sustained Ca2+ misrouting, impaired ATP supply, excessive ROS generation, and collapse of redox homeostasis. This bioenergetic and oxidative failure is followed by defective organelle quality control, particularly insufficient mitophagy, autophagic blockade, lysosomal dysfunction, and ineffective clearance of damaged cellular components. In parallel, mitochondrial injury promotes inflammatory amplification through the release of DAMPs, activation of inflammasome-related pathways, and propagation of sterile inflammatory signaling within the pancreas and beyond. Depending on the severity and reversibility of these interconnected events, PACs may undergo adaptive recovery, apoptosis, or necrotic cell death, whereas extensive and self-sustaining organellar disintegration contributes to tissue injury, systemic inflammation, multiple organ dysfunction, and the severe clinical course of AP.

## 4 Translational relevance: pharmacological modulation of mitochondria–organelle crosstalk in acute pancreatitis

From a translational perspective, the mitochondrial hub model is useful only if it helps distinguish three clinically relevant questions: which events are upstream enough to prevent irreversible injury, which later pathways mainly amplify established damage, and which readouts can be monitored in patients. This approach emphasizes that successful translation in AP will likely require stage-adapted modulation of pathological organelle crosstalk rather than indiscriminate suppression of mitochondrial signaling.

Within this framework, mitochondria are best understood as central interaction hubs for the ER, lysosomes, inflammasome platforms, and other organelles in PACs. In AP, early mitochondrial depolarization, ATP depletion, and opening of the mPTP rapidly feedback on ER Ca^2+^ handling, zymogen activation, autophagic flux, and inflammatory signaling. Experimental data therefore suggest that pharmacological strategies preserving or re-balancing mitochondrial function may normalize several inter-organellar circuits simultaneously, rather than acting on a single linear pathway such as oxidative stress or necrosis. Current experimental approaches can thus be interpreted according to whether they target early mitochondrial injury or later amplifying pathways of organelle dysfunction. One of the best-validated mitochondrial targets in AP is cyclophilin D (CypD), the regulatory component of the mPTP. Genetic deletion or pharmacological inhibition of CypD prevents toxin-induced mitochondrial depolarization, ATP collapse, and necrotic death of acinar cells, and consistently reduces the severity of experimental AP and associated organ failure in rodent models (42, 80, 81). Classical CypD inhibitors, such as cyclosporin A and its non-immunosuppressive analogue NIM811, attenuate mitochondrial Ca^2+^ overload, preserve ΔΨm in both acinar and ductal cells, reduce necrosis, and dampen systemic inflammatory responses. More recently, urea-based and pyrazolidine CypD inhibitors with nanomolar affinity have shown robust protection of pancreatic mitochondria and tissue architecture in cerulein- and bile acid-induced AP, confirming that mPTP inhibition is a druggable node that functionally stabilizes mitochondrial–ER Ca^2+^ crosstalk, ROS generation, and cell-death patterns. These observations place mPTP inhibition among the most upstream pharmacological strategies currently available in experimental AP. However, the translational profile of this class remains complex. Cyclosporin A is clinically available, but its feasibility in AP is limited by a narrow therapeutic index, nephrotoxicity, neurotoxicity, infection risk, and the fact that systemic calcineurin inhibition is poorly suited to a rapidly evolving sterile inflammatory disorder. In addition, cyclosporin exposure has itself been associated with direct exocrine pancreatic injury and with worsening of experimental pancreatitis, which complicates its repositioning for AP (82, 83). NIM811 avoids major immunosuppressive effects and is therefore mechanistically more attractive, but it has not advanced into pancreatitis-specific clinical testing, and there are currently no human trial data showing that CypD blockade improves outcomes in AP. Thus, despite strong biological validation, mPTP inhibition should presently be regarded as a promising preclinical strategy rather than a clinically actionable therapy.

Another key aspect of mitochondria–organelle communication in AP is mitochondrial quality control via mitophagy and its tight coupling to lysosomal function. Severe AP is characterized by impaired autophagic flux with accumulation of large autophagosomes and overloaded lysosomes, leading to the persistence of dysfunctional mitochondria, excess mtROS, and the release of mitochondrial DAMPs. Recent work has identified Sestrin2 (Sesn2) as a central “switch” between mitophagy and mitochondrial apoptosis in experimental severe AP. In an LPS/cerulein model, up-regulation of Sesn2 increased ΔΨm, reduced mitochondrial superoxide production, limited apoptosis, and shifted macrophage polarization toward an anti-inflammatory M2 phenotype by enhancing PINK1–Parkin-dependent mitophagy, whereas Sesn2 knockout aggravated pancreatic and mitochondrial injury (69). Although Sesn2 was manipulated genetically rather than pharmacologically in that study, both Sesn2 itself and its upstream regulators, mTOR and AMPK, are in principle amenable to small-molecule targeting. This positions the Sesn2–PINK1–Parkin axis as an attractive pharmacological entry point to restore integrated mitochondria–lysosome quality control, promote clearance of damaged mitochondria, reduce mtROS and DAMP release, and secondarily limit NLRP3 inflammasome activation and ER stress. Importantly, several core mitophagy regulators are organized at or influenced by mitochondria-associated ER membranes (MAMs), indicating that mitophagy-directed therapy may also act, at least in part, through remodeling of organelle contact sites rather than through mitochondria alone. In this regard, PINK1/Parkin signaling, FUNDC1-related stress responses, DRP1-dependent mitochondrial segregation, and VMP1-linked autophagosome formation represent more mechanistically focused candidate nodes than broad antioxidant or anti-inflammatory interventions, although direct pharmacological evidence in AP remains limited (14, 84). In this context, enhancement of mitophagy is best viewed as a strategy to limit persistence and amplification of mitochondrial injury rather than to block the earliest initiating insult. At the same time, therapeutic stimulation of mitophagy should not be assumed to be uniformly beneficial across all stages of AP. Its potential value is most plausible during phases in which damaged mitochondria can still be efficiently cleared and replaced, whereas in advanced disease characterized by profound ATP depletion, lysosomal dysfunction, and impaired organelle renewal, indiscriminate enhancement of mitochondrial sequestration could theoretically aggravate bioenergetic failure rather than restore homeostasis. Importantly, this concern remains inferential, because current evidence does not directly demonstrate that excessive or prolonged mitophagy worsens pancreatic injury *in vivo*; however, it does argue for cautious stage-specific interpretation of mitophagy-targeted interventions. A further translational limitation is that no selective mitophagy-enhancing drug has yet been validated in patients with AP. Most currently available ways to influence this axis, including AMPK activation or mTOR modulation, are systemic and pleiotropic, so their net effects are unlikely to reflect pancreas-specific restoration of mitochondrial quality control alone. This is particularly relevant in critically ill patients, in whom generalized manipulation of nutrient-sensing and autophagy pathways may produce off-target metabolic, immunological, or hemodynamic consequences.

Given the dominant contribution of oxidative stress to mitochondrial dysfunction in AP, mitochondria-targeted antioxidants have also been investigated as a means to protect mitochondria–organelle crosstalk. The best-known compound, MitoQ, is a ubiquinone linked to a triphenylphosphonium cation, which accumulates within the inner mitochondrial membrane. In isolated PACs, MitoQ and the control cation Decyl-TPP increased basal respiration, but markedly reduced ATP-linked respiration, increased proton leak, and promoted both apoptosis and necrosis in a concentration-dependent manner, reflecting deleterious perturbation of mitochondrial bioenergetics rather than simple antioxidant protection (85). *In vivo*, MitoQ produced mixed or nonprotective effects in AP models. These findings highlight that merely delivering high concentrations of antioxidants to mitochondria is not sufficient and may even be harmful if the carrier moiety disrupts the inner membrane function. In the context of organelle crosstalk, such strategies may destabilize the mitochondrial hub that they are meant to protect, with downstream negative consequences for ER Ca^2+^ signaling, autophagy, and inflammasome assembly. Future mitochondria-directed antioxidants for AP will need to avoid strong membrane-perturbing cations, be optimized specifically for acinar-cell bioenergetics, and be evaluated not only for redox effects but also for their impact on mitochondrial ATP production and Ca^2+^ handling. These data also suggest that targeting oxidative stress alone may be insufficient when upstream mitochondrial energetic failure is not simultaneously controlled. From a clinical perspective, MitoQ is one of the few mitochondria-targeted agents with human safety experience outside pancreatitis, including early-phase clinical evaluation in non-pancreatic settings (86, 87). Nevertheless, this background should not be overinterpreted as disease-specific readiness for AP. The pancreatic preclinical data raise a clear caution that a compound considered systemically tolerable may still be maladaptive in acinar cells if mitochondrial membrane accumulation disrupts respiration. Accordingly, AP translation will require not only conventional toxicology but also pancreas-relevant assessment of carrier chemistry, dosing, and timing relative to the onset of mitochondrial depolarization.

Mitochondria are also closely linked to inflammatory signaling through the NLRP3 inflammasome. The activation of NLRP3 in AP depends on mtROS, oxidized cardiolipin, mitochondrial DNA released from damaged mitochondria, ion fluxes, and lysosomal signals. Deletion or pharmacological inhibition of NLRP3 reduces pancreatic necrosis, cytokine release, and distant organ injury in several AP models (88, 89). The small-molecule NLRP3 inhibitor MCC950 shows strong protection in rodent models of AP by decreasing IL-1β maturation, neutrophil infiltration, and multi-organ dysfunction, although its clinical translation has been hampered by off-target toxicity and resistance of certain NLRP3 conformers. A more recent study identified the natural stilbenoid polydatin as an inhibitor of HSP90α that destabilizes both resting and active NLRP3 complexes, thereby suppressing inflammasome assembly and significantly attenuating cerulein-induced AP (90). Polydatin does not act via direct binding to NLRP3, but rather by disrupting its chaperone-mediated stabilization, providing an alternative way to interfere with NLRP3–mitochondria signaling platforms. Other phytochemicals, including proanthocyanidins and naringenin, have been reported to suppress NLRP3 activation, decrease mtROS, and favor anti-inflammatory macrophage polarization in AP, again acting functionally at the interface between mitochondrial redox status, lysosomal integrity, and inflammasome assembly. Even if their primary molecular targets are not strictly mitochondrial, these compounds modulate mitochondria–inflammasome crosstalk by attenuating downstream consequences of mitochondrial distress. Accordingly, inflammasome-directed interventions mainly target downstream inflammatory amplification rather than the earliest mitochondrial lesion. This distinction is relevant when considering mitophagy-oriented strategies, because beneficial effects observed after suppression of downstream inflammatory mediators do not by themselves establish that direct augmentation of mitophagy in PACs would remain advantageous under all pathological conditions. Their translational attractiveness is nevertheless tempered by several practical issues. MCC950 has not entered clinical use for AP, and broader development of this chemical class has been constrained by safety concerns, particularly hepatotoxicity signals observed during clinical development in other inflammatory settings (91, 92). This issue is especially relevant in AP, where biliary obstruction, cholestasis, or secondary liver injury may already coexist. Natural compounds such as polydatin, naringenin, or proanthocyanidins are pharmacologically appealing because of their multi-target anti-inflammatory effects, but they also face familiar barriers of low oral bioavailability, uncertain pancreas exposure, and limited pharmacokinetic standardization. Thus, current inflammasome-directed approaches in AP remain biologically informative but clinically immature.

A further pharmacological layer involves systemic regulators of mitochondrial biogenesis and stress responses that indirectly influence several organelle contact-dependent processes. Compounds such as resveratrol and other SIRT1 activators have shown beneficial effects in AP models by improving mitochondrial respiration, limiting oxidative stress, and attenuating ER stress-mediated apoptosis (93). Through activation of the SIRT1–PGC-1α axis and related pathways, these agents are likely to influence mitochondrial–ER Ca^2+^ communication, β-oxidation in cooperation with peroxisomes, and the cellular capacity to cope with redox stress; however, direct evidence for their effects at defined organelle contact sites, such as MAMs, in AP remains limited. In addition, proteasome and HSP90 inhibitors can affect the stability of client proteins involved in mitochondrial dynamics, inflammasome assembly, and fibrotic remodeling of the pancreas, suggesting additional, although less specific, ways to influence mitochondria–organelle networks. Because many of these approaches act systemically and influence multiple stress-response pathways simultaneously, their beneficial effects cannot be interpreted as selective proof that a PAC-specific increase in mitophagy is itself sufficient or universally desirable. This limitation applies particularly to pharmacological agents that modify AMPK, mTOR, redox signaling, or inflammatory cascades in parallel with autophagy-related processes. The same caution applies to other compounds discussed in the context of mitochondrial protection, including matrine, pinocembrin, α-lipoic acid, and related pleiotropic agents: although they may improve mitochondrial performance and attenuate ER stress, inflammation, or redox imbalance, they should be regarded primarily as broad stress-response modulators rather than selective regulators of organelle contact sites. By contrast, genuinely contact-site-oriented strategies would aim to modulate defined MAM-tethering or signaling complexes, such as the IP_3_R–GRP75–VDAC1 Ca^2+^ transfer axis, MFN2-dependent ER–mitochondria juxtaposition, or the VAPB–PTPIP51 tethering interface, all of which are increasingly recognized as major determinants of Ca^2+^ exchange, bioenergetics, and mitophagy initiation in stressed cells (84, 94). However, no selective pharmacological modulator of these MAM nodes has yet been validated in AP, and even in other fields most available interventions remain indirect, context-dependent, or genetically defined rather than clinically deployable small molecules. In practical terms, the field still lacks agents with a clearly developed medicinal-chemistry trajectory, pancreas-restricted delivery, and a safety profile suitable for emergency administration in patients who often present with hypovolemia, evolving organ dysfunction, and marked inter-individual variability in etiology and severity.

This translational distinction is crucial. The immediate value of the mitochondrial hub framework is not that it already delivers a bedside therapy, but that it prioritizes which nodes are most actionable, when they should be targeted, and how benefit should be measured. In practical terms, the near-term translational agenda includes three linked goals: development of stage-aware biomarkers of mitochondrial injury and network failure, including circulating mitochondrial-damage signals; testing therapeutically timed interventions in etiology-diverse and human-relevant models; and creation of delivery strategies that dampen pathological contact-site remodeling without abolishing the physiological organelle coupling required for secretion and recovery. Such selectivity remains challenging because organelle contacts are dynamic, cell-type-specific, and indispensable for normal pancreatic function. These considerations make it unlikely that successful clinical translation will come from complete pharmacological shutdown of organelle communication; a more realistic objective is partial, stage-adapted stabilization of pathological remodeling while preserving the physiological architecture of the acinar cell.

Taken together, current evidence indicates that mitochondria–organelle crosstalk is a rational therapeutic theme in AP, but still a preclinical one. The most credible near-term targets are upstream regulators of mitochondrial depolarization and Ca^2+^ overload, whereas modulation of mitophagy, inflammasome signaling, and redox stress may be better suited to limiting secondary injury amplification. Future studies should therefore prioritize therapeutic rather than prophylactic dosing, inducible and cell-restricted designs, etiologically diverse models beyond caerulein paradigms, and validation in human acinar, organoid, or ex vivo platforms. Only under these conditions will it be possible to determine whether selective stabilization of the mitochondrial hub can move from mechanistic plausibility to clinically meaningful intervention (95).

A practical translational roadmap emerges from this framework. For early stratification, future studies should test panels that combine conventional severity assessment with circulating indicators of mitochondrial injury and organelle-network stress, particularly mitochondrial DNA release and related inflammatory readouts detectable in patients (99). For intervention studies, the most informative designs will be those that pair such biomarkers with therapeutically timed treatment in human acinar, organoid, or ex vivo tissue platforms before progression to etiology-diverse *in vivo* models (96). Such a staged program would help determine not only whether a compound is protective, but also whether it acts by stabilizing an upstream contact-site defect, limiting secondary amplification, or merely delaying terminal bioenergetic failure.

## 5 Unresolved issues and future research directions

Despite substantial progress in understanding inter-organellar interactions in PACs, several key aspects remain insufficiently explored. One of the major unresolved issues is the temporal and mechanistic hierarchy of organelle dysfunction in AP, particularly the relative sequence linking Ca^2+^ overload, mitochondrial depolarization, bioenergetic failure, oxidative stress, defective mitophagy, inflammasome activation, and necrotic cell death. An additional unresolved question is whether mitophagy exerts a uniformly protective effect throughout the course of AP or whether its impact is stage-dependent. Available experimental data support a predominantly adaptive role in the early phase, when elimination of depolarized mitochondria may limit mtROS generation, DAMP release, and inflammatory amplification (35, 69–72). However, in later stages of severe disease, when ATP depletion, lysosomal dysfunction, impaired mitochondrial replenishment, and widespread necrosis are already established, prolonged or excessive mitophagic activity could theoretically become maladaptive if mitochondrial clearance exceeds the capacity for mitochondrial replacement and thereby aggravates bioenergetic failure. Further studies are needed to elucidate the mechanisms of formation and regulation of mitochondria–ER contacts, their plasticity in response to stress stimuli, and the molecular determinants that preserve MAM integrity under pathological conditions. The transport of organelles along the cytoskeleton and the role of mitochondrial dynamics in modulating autophagic flux also remain poorly understood. Another promising area involves the investigation of retrograde signaling between mitochondria and the nucleus, which may regulate transcriptional programs of inflammation and repair. In particular, it remains unclear which of these processes act predominantly as early drivers of acinar cell injury and which function mainly as secondary amplifiers that sustain local and systemic inflammation. At present, direct proof that excessive or prolonged mitophagy itself causes energy depletion in PACs remains limited, because most available studies rely on static endpoints or constitutive interventions rather than time-resolved measurements integrating mitophagic flux, mitochondrial biogenesis, and ATP recovery (35, 69–72). Future research should be expanded using advanced super-resolution microscopy, cellular bioenergetics approaches, and metabolomics. Longitudinal and pancreas-specific studies combining real-time imaging, organelle-selective reporters, and time-resolved functional assays will be particularly important for resolving these relationships. Such designs will also be essential for distinguishing PAC-intrinsic effects of mitophagy from indirect consequences mediated by inflammatory cells, endothelial injury, or systemic metabolic stress. It is also important to explore potential therapeutic strategies aimed at restoring inter-organellar integration through small molecules, redox-signaling modulators, and inhibitors of calcium overload. In this context, interpretation of current mechanistic studies should remain cautious, because many conclusions regarding mitophagy are derived from global genetic manipulations or systemic pharmacological interventions that influence multiple cell types and several autophagy-related pathways simultaneously, making PAC-specific attribution difficult (35, 69–72). A major priority for future research is the direct validation of the proposed organelle crosstalk model in human AP. At present, the available human evidence is supportive but incomplete. Proteomic support is provided by ex vivo studies of human PACs demonstrating that human acini preserve key physiological properties while developing pancreatitis-relevant stress responses and organellar abnormalities, indicating that several mechanistic features identified in rodents are also detectable in human acinar tissue (96). Histological and ultrastructural human data also support the presence of mitochondrial injury, ER alterations, and intracellular vacuolization in the exocrine pancreas, although these observations remain largely descriptive and do not resolve dynamic remodeling of specific contact sites such as ER–mitochondria or mitochondria–lysosome interfaces (97). Transcriptomic support is currently more limited and indirect, because the available single-cell RNA sequencing datasets in human severe AP are derived predominantly from peripheral blood immune cells and therefore reflect systemic inflammatory activation rather than pancreas-specific inter-organellar signaling within acinar cells (98). In addition, clinical studies showing an association between circulating mitochondrial DNA and disease severity provide further evidence that mitochondrial injury is relevant in human AP, but they do not directly validate the hierarchical remodeling of inter-organellar networks in pancreatic tissue (99). Thus, future progress will require integrated studies based on human pancreatic tissue, spatial and single-cell transcriptomics, organelle-oriented proteomics, advanced imaging, and correlative ultrastructural analysis in well-characterized patient cohorts in order to determine to what extent the mitochondrial hub model established in experimental systems is conserved in human disease.

## 6 Limitations

These conclusions rely predominantly on experimental studies in rodents and isolated PACs exposed to supramaximal secretagogue stimulation or toxin-induced injury (**Table 3**). The resulting evidence base is mechanistically informative but inherently reductionist. These models only partially reproduce the complexity of human AP, in which biliary, alcoholic, metabolic, and drug-related triggers coexist with comorbidities such as obesity, diabetes, and chronic alcohol use. In addition, the clinical syndrome evolves in the context of variable delays in presentation, fluid resuscitation, organ-support measures, infection risk, and pre-existing systemic vulnerability, all of which are difficult to model faithfully in standard experimental settings. Most mechanistic insights into mitochondria–ER, mitochondria–lysosome, and other organelle contacts have been derived from indirect functional readouts or static ultrastructural analyses. Dynamic, high-resolution *in vivo* imaging of contact sites in the inflamed pancreas is virtually absent, making temporal and causal relationships between organelle remodeling, calcium mishandling, bioenergetic failure, and cell death largely inferential. As a result, the distinction between early initiating events and secondary amplifying processes remains difficult to define with precision. Furthermore, many key tethering complexes and signaling pathways have been dissected in non-pancreatic systems, such as neurons or cancer cell lines, and then extrapolated to PACs without systematic validation of tissue-specific features. This limitation is particularly relevant to lipid droplet–mitochondria interactions, because specific tethering assemblies such as PLIN5–FATP4–MIGA2–MFN2 have not yet been directly demonstrated in PACs, and their proposed role in the pancreas is therefore extrapolated from other metabolically active cell types. In addition, the available data sets are heterogeneous with respect to species, age, sex, injury models, experimental endpoints, and quantification methods. This heterogeneity hinders direct comparisons across studies and precludes quantitative synthesis. Many studies also rely on single or limited time-point sampling, pharmacological inhibition with pleiotropic effects, or global genetic models, all of which further constrain interpretation of temporal sequence and cell-specific causality. A further methodological concern is that widely used models, particularly caerulein-based paradigms, capture synchronized acinar-cell stress and early mitochondrial injury very effectively, but they incompletely reproduce the full spectrum of necrotizing, biliary, hypertriglyceridemic, post-ERCP, or alcohol-associated human disease. Consequently, efficacy observed in these systems should primarily be interpreted as proof of biological plausibility and mechanism, rather than as a direct surrogate of clinical efficacy. Finally, translational implications remain speculative because most interventions targeting mitochondrial or inter-organellar functions have been tested in prophylactic or early-treatment settings in animals, with little evidence regarding their efficacy, safety, and feasibility in established, severe disease in humans. This issue is especially important for therapies directed at mitochondria–organelle crosstalk, because the balance between reversible stress adaptation and irreversible bioenergetic collapse is strongly time-dependent, and the therapeutic window in patients is often substantially later than in experimental studies.

**Table 3:** Experimental models of acute pancreatitis and mitochondrial/organelle endpoints.

Model/species	Method of AP induction	Main mitochondrial readouts	Organelle interactions specifically assessed	Key findings related to mitochondrial–organelle crosstalk
Mouse/rat *in vivo*; isolated acinar cells ex vivo	Supramaximal caerulein stimulation (with or without LPS)	ΔΨm, ATP content, mitochondrial Ca^2+^, ROS generation, mPTP opening	Mitochondria–ER contacts (Ca^2+^ transfer via MAMs); coupling to zymogen granules and secretory machinery	Supramaximal stimulation causes sustained cytosolic and mitochondrial Ca^2+^ overload, collapse of ΔΨm, ATP depletion, and necrosis; disruption of ER–mitochondria Ca^2+^ handling and polarized secretion; central role of mitochondrial failure in early AP
Mouse *in vivo*; acinar cells *in vitro*	Bile acid–induced AP (e.g., taurolithocholic acid sulfate, TLCS)	Mitochondrial Ca^2+^, ΔΨm, ROS, cell death type (necrosis vs apoptosis)	ER–mitochondria Ca^2+^ microdomains; interaction with plasma membrane Ca^2+^ entry	Bile acids cause rapid Ca^2+^ influx and ER release, leading to mitochondrial Ca^2+^ overload via MAMs; early ΔΨm dissipation and necrosis; mitochondria act as core integrators of bile acid toxicity in AP
Mouse *in vivo*; acinar cells ex vivo	Alcohol/FAEE-related models (ethanol + fatty acids; FAEE administration)	ΔΨm, ROS, mitochondrial Ca^2+^, lipid accumulation	Mitochondria–lipid droplet contacts; interaction with ER and peroxisomes	FAEEs and long-chain fatty acids induce mitochondrial depolarization, Ca^2+^ dysregulation, ROS overproduction, and lipotoxicity; mitochondria–lipid droplet interactions convert metabolic stress into acinar cell necrosis, contributing to alcoholic and hypertriglyceridemic AP
Mouse *in vivo*	L-arginine–induced necrotizing AP	ΔΨm, mitochondrial swelling, ATP levels, oxidative stress markers	Global mitochondrial network integrity; interaction with lysosomes and autophagy machinery	High-dose L-arginine causes severe mitochondrial damage, swelling, and depolarization, with profound ATP depletion; defective autophagy/mitophagy and accumulation of damaged mitochondria correlate with extensive necrosis and systemic toxicity
Mouse *in vivo*; acinar cells ex vivo	Combined inflammatory and toxic models (e.g., caerulein + LPS; caerulein + ethanol/fatty acids)	ΔΨm, ROS, Ca^2+^, mtDAMP release, inflammasome activation	Mitochondrial communication with nucleus (inflammatory transcription), lysosomes (mitophagy), and immune cells	Synergistic insults exaggerate mitochondrial dysfunction, ROS production, and mtDAMP release; drive strong activation of inflammatory pathways (e.g., NLRP3) and systemic organ injury, mimicking severe human AP
Isolated pancreatic acinar cells (mouse/rat) *in vitro*	Pharmacological modulation of Ca^2+^ channels, mPTP, autophagy, mitochondrial dynamics	High-resolution imaging of mitochondrial morphology, Ca^2+^, ΔΨm, autophagosomes; targeted genetic or pharmacological interventions	Detailed analysis of ER–mitochondria Ca²^+^ coupling, mitochondria–lysosome contacts, and mitochondrial dynamics	Selective inhibition or enhancement of individual pathways (e.g., SOCE, mPTP, DRP1-mediated fission, mitophagy) demonstrates causal links between specific mitochondrial–organelle interactions and acinar cell fate, validating the “mitochondrial hub” concept in AP

An additional major limitation is that direct human evidence supporting the proposed organelle crosstalk framework remains fragmented. Although human proteomic data from ex vivo acinar-cell preparations support the relevance of stress-associated organellar injury in pancreatitis-relevant conditions, these systems do not reproduce the full multicellular, vascular, stromal, and immune complexity of clinical AP (96). Human histological and ultrastructural datasets also demonstrate mitochondrial abnormalities, ER injury, and vacuolization in the human exocrine pancreas, but these findings are largely descriptive and do not establish causal or dynamic relationships between specific organelle contact sites (97). Likewise, the currently available human transcriptomic datasets provide only indirect support for the proposed model, because they are predominantly based on peripheral blood immune-cell profiling and therefore capture systemic inflammatory responses rather than direct pancreas-specific remodeling of mitochondrial, ER, lysosomal, or secretory-granule interactions in acinar cells (98). Clinical biomarker data, including the association of circulating mitochondrial DNA with disease severity and pancreatic necrosis, further support the relevance of mitochondrial damage in patients, but again do not directly validate the structural and functional hierarchy of the inter-organellar network proposed from experimental studies (99). Moreover, direct access to early human pancreatic tissue is exceptionally limited for ethical and practical reasons, so the earliest phases of organelle disorganization, Ca^2+^ dysregulation, and mitochondrial failure cannot usually be studied at the stage when they are most likely to be mechanistically informative. Human material is therefore often obtained late, indirectly, or under highly selected ex vivo conditions, which introduces a substantial sampling bias toward advanced injury or incomplete tissue context.

Accordingly, the major translational gaps between experimental models and human disease include species-specific differences in acinar-cell physiology and calcium handling, the oversimplified nature of experimental triggers compared with the etiological heterogeneity of clinical pancreatitis, the absence of common human comorbid modifiers in many animal models, limited access to early human pancreatic tissue for direct mechanistic analysis, and the lack of integrated human datasets linking morphology, transcriptomics, proteomics, and functional mapping of organelle interactions. Another major gap is the absence of longitudinal human studies capable of connecting early mitochondrial dysfunction to subsequent ER failure, lysosomal collapse, inflammasome activation, and clinical progression within the same patients. Without this type of integrated temporal mapping, the hierarchy of inter-organellar events proposed from experimental systems remains biologically plausible but not definitively validated in humans. Another important gap is that most animal studies assess preventive or very early interventions, whereas patients usually present after organelle injury, inflammation, and systemic complications are already established. This discrepancy limits confidence not only in efficacy estimates but also in safety and feasibility assumptions, because compounds that appear beneficial in preventive rodent paradigms may behave differently when administered later in the course of severe disease, in the presence of hypoperfusion, cholestasis, renal dysfunction, or evolving multi-organ failure. These limitations should temper direct extrapolation of experimentally defined mechanistic hierarchies to human AP. Future progress will depend on combining refined animal models with therapeutic, rather than exclusively preventive, intervention designs; broader use of human acinar, organoid, and ex vivo platforms; and the development of multimodal human datasets integrating ultrastructural, molecular, and functional readouts of organelle interactions. Until such evidence becomes available, mitochondria–organelle crosstalk should be regarded as a strong integrative framework for AP pathobiology, but not yet as a fully validated map of causal hierarchy in human disease.

## 7 Conclusions

Mitochondria in PACs should be viewed as an inter-organellar hub that integrates Ca^2+^ signaling, bioenergetics, redox control, organelle quality control, and inflammatory signaling through dynamic contacts with the ER, lysosomes/autophagosomes, peroxisomes, lipid droplets, the cytoskeleton, plasma membrane, nucleus, and secretory compartment. In AP, failure of this hub converts spatially restricted stress responses into self-amplifying network collapse, marked by pathological Ca2+ transfer, mPTP opening, ATP depletion, oxidative and lipotoxic injury, defective clearance of damaged organelles, and necroinflammatory escalation.

These events as a coordinated hierarchy rather than as parallel abnormalities. This perspective explains why early ER–mitochondria Ca^2+^ maladaptation, later lysosomal-autophagic failure, redox amplification, inflammasome activation, and cell-fate decisions are mechanistically linked, and it generates testable predictions about temporal ordering, reversibility, and disease-stage specificity of contact-site remodeling. The translational relevance of this framework lies in its ability to guide biomarker discovery, model selection, and therapeutic prioritization. It supports the development of stage-aware biomarkers of mitochondrial distress and contact-site failure, encourages use of human-relevant and therapeutically timed experimental systems, and identifies selective stabilization of pathological organelle crosstalk as a more realistic goal than global suppression of mitochondrial signaling. Equally important, it sets realistic expectations for the field: at present, the mitochondrial hub model is most useful for ranking targets, defining therapeutic windows, and structuring translational studies, whereas direct clinical intervention remains investigational. Accordingly, the mitochondrial hub model should be regarded as a useful, mechanistically grounded framework for future experimental and translational research in acute pancreatitis.
